# HBXIP induces anoikis resistance by forming a reciprocal feedback loop with Nrf2 to maintain redox homeostasis and stabilize Prdx1 in breast cancer

**DOI:** 10.1038/s41523-021-00374-x

**Published:** 2022-01-13

**Authors:** Xiaolei Zhou, Li Li, Xin Guo, Chunxiao Zhang, Yanyan Du, Tianming Li, Kaiqing Tong, Chongyue Zhu, Zijin Wang

**Affiliations:** 1 College of Bioscience & Bioengineering, Hebei University of Science and Technology, Shijiazhuang, 050018 PR China; 2 Department of ICU, Shijiazhuang Fifth Hospital, Shijiazhuang, 050021 PR China; 3 Department of Pathology and Laboratory Medicine, Kanazawa Medical University, 1-1 Daigaku, Uchinada, Ishikawa Japan; 4 Department of Pathology, Kanazawa Medical University Hospital, 1-1 Daigaku, Uchinada, Ishikawa Japan; 5 Department of Clinical Laboratory, The Fourth Hospital of Hebei Medical University, Shijiazhuang, 050031 PR China

**Keywords:** Apoptosis, Breast cancer, Oncogenes

## Abstract

Anoikis resistance is an essential prerequisite for tumor metastasis, but the underlying molecular mechanisms remain unknown. Herein, we report that the oncoprotein hepatitis B X-interacting protein (HBXIP) is prominently upregulated in breast cancer cells following ECM detachment. Altering HBXIP expression can impair the anchorage-independent growth ability of tumor cells. Mechanistically, HBXIP, which binds to Kelch-like ECH-associated protein 1 (Keap1) to activate nuclear factor E2-related factor 2 (Nrf2), contains a cis-acting antioxidant response element (ARE) in the gene promoter and is a target gene of Nrf2. The HBXIP/Nrf2 axis forms a reciprocal positive feedback loop that reinforces the expression and tumor-promoting actions of each protein. In response to ECM detachment, Nrf2 reduces reactive oxygen species (ROS) accumulation, protects the mitochondrial membrane potential and increases cellular ATP, GSH and NADPH levels to maintain breast cancer cell survival. Meanwhile, the reinforcement of HBXIP induced by Nrf2 inhibits JNK1 activation by inhibiting ubiquitin-mediated degradation of Prdx1, which also plays an essential role in promoting ECM-detached cell survival. Furthermore, a strong positive correlation was identified between HBXIP expression and Prdx1 expression in clinical breast cancer tissues and TCGA Pan-Cancer Atlas clinical data of breast invasive carcinoma based on the cBioPortal cancer genomics database. Co-expression of HBXIP and Prdx1 predicts a poor prognosis for breast cancer patients. Collectively, our findings reveal a significant mechanism by which the HBXIP/Nrf2 feedback loop contributes to anoikis resistance by maintaining redox homeostasis and inhibiting JNK1 activation and support the likely therapeutic value of the HBXIP/Nrf2 axis in breast cancer patients.

## Introduction

Breast cancer metastasis is a multi-step and multi-factor process, including the reintegration and degradation of the extracellular matrix (ECM), detachment from local tissue, invasion of blood or the lymphatic system, and the formation of new tumors in distant locations. Loss of attachment or inappropriate attachment to the ECM and neighboring cells results in programmed cell death, referred to as anoikis. Studies have confirmed that anoikis serves as the first line of defense against metastasis and is an early intervention preventing cancer metastasis^1^. Anoikis resistance endows malignant breast cancer cells with anchorage-independent growth abilities, which aid in tumor metastasis. Tumor cells adopt multilevel strategies to evade anoikis, such as alterations in glucose and fatty acid metabolism^2^, reactive oxygen species (ROS)-mediated activation of signaling pathways^3^, alterations in autophagy signaling^4^, integrin deregulation^5^, and aberrant constitutive activation of several antiapoptotic or prosurvival pathways^1^. Despite these important findings from previous studies, the large number of unknown mechanisms involved in anoikis resistance remains a complex problem requiring further research.

Mammalian hepatitis B X-interacting protein (HBXIP, also termed LAMTOR5) is an 18.6-kDa protein that is highly conserved among mammals. Studies have confirmed that HBXIP is an oncoprotein that is markedly enriched in cancer tissues^6,7^. HBXIP functions as an oncogenic transcriptional coactivator of various transcription factors (TFs), such as SP1, TF-IID, STAT3, CREB, and E2F1, in the promotion of breast cancer tumorigenesis and progression^6–8^. Recently, our group revealed a regulatory mechanism for cellular ROS production and the progression of breast cancer in which HBXIP functions as a critical modulator of the classical Keap-Nrf2-antioxidant response element (ARE) pathway^9^. HBXIP effectively competes with Nrf2 for binding to the Keap1 protein and promotes the nuclear entry and phosphorylation of Nrf2, which modulates the activation of ARE-dependent signaling pathways, thereby reducing cellular ROS levels^9^. However, whether HBXIP is involved in breast cancer cell anoikis resistance remains unclear.

In the present study, we provided the first evidence that HBXIP is upregulated and forms a reciprocal HBXIP/Nrf2 feedback loop in breast cancer cells to promote cell survival following ECM detachment. Mechanistically, the *HBXIP* gene contains a cis-acting ARE in its promoter that can be recognized and activated by Nrf2. Furthermore, the reinforced HBXIP effectively competes with Nrf2 for binding to the Keap1 protein^9^ and activates the Nrf2-ARE pathway. The positive HBXIP/Nrf2 feedback loop promotes the anchorage-independent survival of cancer cells through Nrf2 maintenance of robust ROS levels and HBXIP stabilization of peroxiredoxin 1 (Prdx1) to inhibit JNK activation. Taken together, our findings provide insight into the molecular mechanism modulating breast cancer cell anoikis and metastasis and offer the probable therapeutic value of the HBXIP/Nrf2 axis in breast cancer patients.

## Results

### HBXIP is upregulated following ECM detachment and induces anoikis resistance of breast cancer cells

**Fig. 1:**
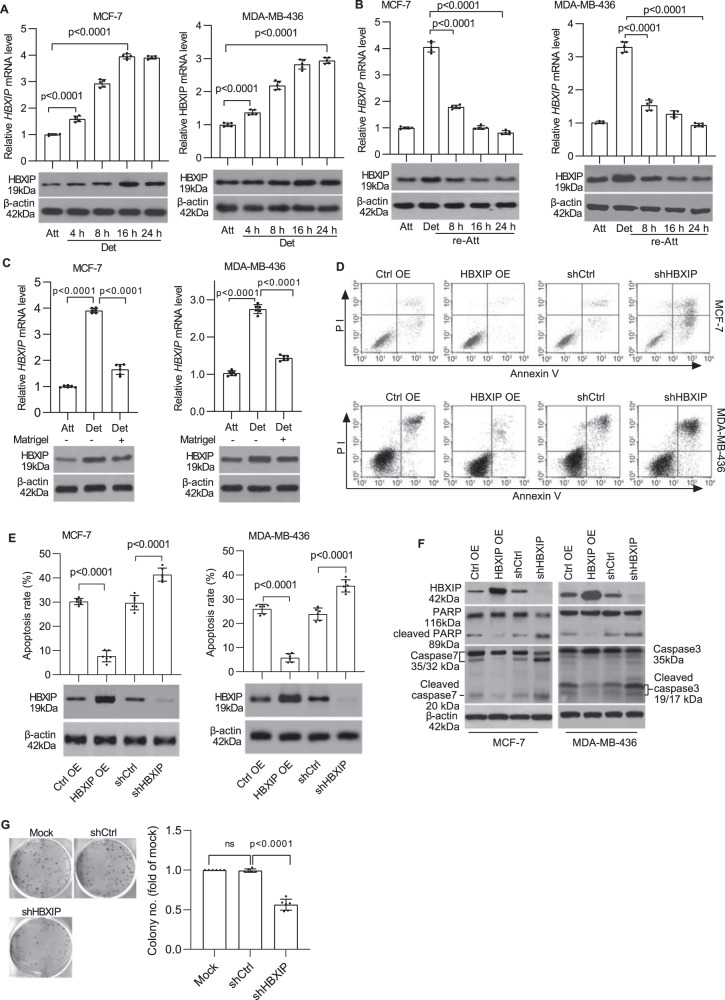
HBXIP is upregulated following ECM detachment and induces anoikis resistance in breast cancer cells. **A** MCF-7 and MDA-MB-436 cells were detached and cultured in suspension in poly-HEMA-coated plates for the indicated times. HBXIP mRNA and protein levels were measured using qRT-PCR (upper panel) and western blot analysis (lower panel), respectively. **B** MCF-7 and MDA-MB-436 cells cultured in suspension for 8 h were reattached for the indicated times and subsequently subjected to qRT-PCR and immunoblot analysis to determine the mRNA and protein levels of HBXIP. **C** qRT-PCR and immunoblot analysis of the HBXIP mRNA and protein expression levels in attached cells and detached cells cultured in complete medium with or without a Matrigel basement membrane-like matrix for 12 h. **D** The stable HBXIP overexpression and knockdown cells were cultured in suspension for 24 h and stained with Annexin V. The flow cytometry profile shows Annexin V-FITC staining on the x-axis and PI staining on the y-axis. FSC/SSC plot and gating strategies were shown in Supplementary Fig. 4. **E** Histograms indicating the percentages of apoptotic cells (early and late apoptosis) in **D** under the indicated conditions. Immunoblots showing HBXIP expression in the indicated stable cell lines. **F** Western blot analysis was performed to detect caspase-3 (or caspase-7), PARP, and cleaved caspase-3 (or cleaved caspase-7) and PARP levels in the stable cell lines described in **D**. **G** A soft agar colony formation experiment was performed to detect the anchorage-independent growth of HBXIP-deficient MDA-MB-436 cells. The error bars indicate the ±SD values as assessed by Student’s *t* test. All experiments were performed at least three times.

To clarify the relationship between HBXIP and anoikis in breast cancer cells, we first evaluated the variations in HBXIP expression in MCF-7 (which have relatively low HBXIP expression levels) and MDA-MB-436 (which have relatively high HBXIP expression levels) cells following ECM detachment (Supplementary Fig. 1A). The qRT-PCR and western blot results showed noticeable increases in the levels of both the HBXIP mRNA and protein in a time-dependent manner following detachment (Fig. 1A). The induction of ECM reattachment of suspended breast cancer cells led to decreased HBXIP expression (Fig. 1B). Moreover, the addition of a Matrigel basement membrane-like matrix to breast cancer cells in suspension culture significantly blocked the increases in HBXIP mRNA and protein levels (Fig. 1C). In addition, noticeable increases in the HBXIP protein level were detected in Luminal type T47D, Her2+ type SK-BR3, and TNBC type MDA-MB-231 breast cancer cells following detachment (Supplementary Fig. 1B). These data suggest that HBXIP may be involved in anoikis regulation in breast cancer.

To determine whether HBXIP participates in anoikis in breast cancer cells, we established stable overexpression or knockdown of HBXIP via the ectopic expression of HBXIP cDNA or HBXIP shRNA using lentiviral technology. HBXIP overexpression significantly increased the ratio of ECM-detached living cells, whereas HBXIP deficiency exacerbated the proapoptotic effect of ECM detachment on breast cancer cells (Fig. 1D, E). Enforced HBXIP expression led to a reduction in the cleaved PARP and activated caspase-3 (or caspase-7 in MCF-7 cells) levels in breast cancer cells in suspension culture compared with levels in control group cells. Inversely, significant increases in the levels of cleaved caspase-3 (or caspase-7 in MCF-7 cells) and cleaved PARP in the cell extracts were observed in HBXIP-deficient cells (Fig. 1F). Moreover, HBXIP knockdown significantly suppressed the anchorage-independent growth of MDA-MB-436 cells (Fig. 1G). Previous studies have certified that HBXIP overexpression (or knockdown) potently enhances (or attenuates) the colony formation capacity of various types of cancer cells^9–13^. Taken together, these results indicate that HBXIP induces anoikis resistance in breast cancer cells in vitro.

### Identification of Nrf2 as a positive regulator of the *HBXIP* gene

**Fig. 2:**
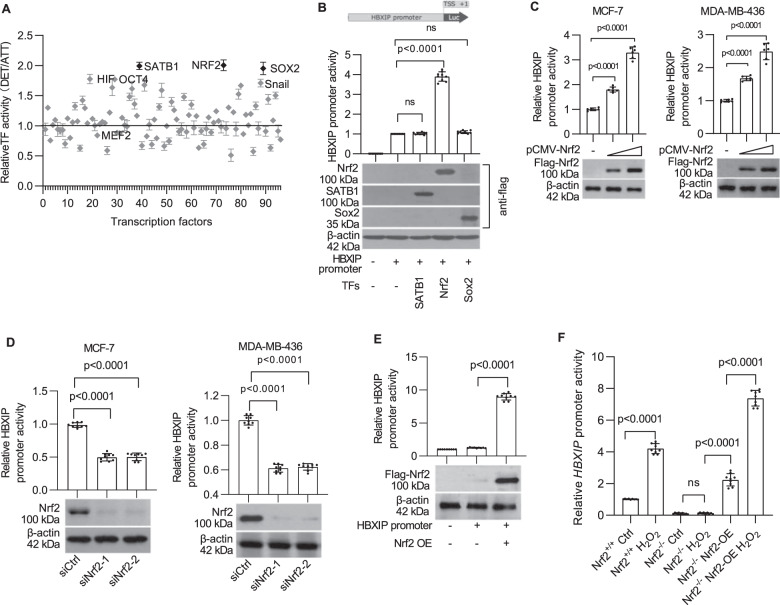
Identification of Nrf2 as a positive regulator of the *HBXIP* gene. **A** The transcription factor profiling assay identified TFs whose activity was altered in MCF-7 cells in response to detachment. **B** *HBXIP* promoter activity was investigated in TF-overexpressing MCF-7 cells. pGL-*HBXIP* FL promoter (from nt -2156 to +357, +1 from the TSS) reporter constructs were separately co-transfected with SATB1, Nrf2 and Sox2 expression vectors. The western blot shows SATB1, Nrf2, and Sox2 expression in transfected MCF-7 cells. **C** *HBXIP* promoter activity was investigated in MCF-7 cells and MDA-MB-436 cells co-transfected with pGL-HBXIP FL promoter-reporter constructs and increasing concentrations of the pCMV-Nrf2 plasmid. Nrf2 expression was detected by western blotting using an anti-Flag antibody. **D** *HBXIP* promoter activity in MCF-7 and MDA-MB-436 cells with Nrf2 knockdown. Nrf2 knockdown by two siRNAs was confirmed by western blotting. **E** *HBXIP* promoter activity was compared between Nrf2-overexpressing HEK293 cells and control cells. Nrf2 expression was confirmed by western blotting using an anti-Flag antibody. **F** Nrf2^−/−^ MEFs, Nrf2^+/+^ MEFs, and human Nrf2-rescued Nrf2^−/−^ MEFs transfected with the pGL-HBXIP FL promoter-reporter plasmid were exposed to 50 μM H_2_O_2_ for 24 h and subjected to a dual-luciferase reporter activity assay. The error bars indicate the ±SD values as assessed by Student’s *t* test. All experiments were performed at least three times.

Next, a pGL3-HBXIP firefly luciferase reporter gene system was established by cloning the ~2.5-kb *HBXIP* promoter sequence amplified from MCF-7 cell chromatin into the vector pGL3. The HBXIP promoter activity increased significantly after cell detachment (Supplementary Fig. 1C), suggesting that certain transcriptional regulators are involved in this process. A transcription factor (TF) activation profiling array assay was conducted using MCF-7 cells cultured under standard and suspension conditions to conclusively determine the mechanism by which breast cancer cells induce *HBXIP* gene transcription. The activities of 96 cellular TFs, such as NF-κB, HIF, and SATB1, which are crucial to the regulation of gene expression^14^, were simultaneously monitored using a TF profiling array. The activity of 5 of the 96 TFs in MCF-7 cells, namely, HIF, OCT4, SATB1, Nrf2, Sox2, and Snail, was increased more than 1.7-fold upon ECM detachment (Fig. 2A). Subsequently, the *HBXIP* promoter activity was verified in MCF-7 cells overexpressing one of the top three potential candidates: SATB1, Nrf2, and Sox2. As shown, Nrf2 but not SATB1 or Sox2 overexpression enhanced *HBXIP* promoter activity (Fig. 2B). Moreover, transfection of increasing concentrations of pCMV-Nrf2 led to a dose-dependent enhancement of *HBXIP* promoter activity in both MCF-7 and MDA-MB-436 cells (Fig. 2C). Conversely, knockdown of Nrf2 by specific siRNAs attenuated *HBXIP* promoter activity (Fig. 2D). These results were also verified in HEK293 cells, which express extremely low levels of endogenous Nrf2 and HBXIP. Coincidentally, enforced Nrf2 expression potently enhanced *HBXIP* promoter activity in HEK293 cells (Fig. 2E). Subsequently, we verified the specificity of Nrf2-mediated activation of *HBXIP* expression using Nrf2^−/−^ mouse embryonic fibroblasts (MEFs) treated with H_2_O_2_, which can activate Nrf2. Reporter gene assays showed that *HBXIP* promoter activity in Nrf2^−/−^ MEFs was potently attenuated relative to that in Nrf2^+/+^ MEFs. In addition, the rescue of Nrf2 expression effectively restored *HBXIP* promoter activity. Furthermore, H_2_O_2_ treatment enhanced *HBXIP* promoter activity in Nrf2^+/+^ MEFs and in rescued Nrf2^−/−^ MEFs (Fig. 2F and Supplementary Fig. 1D). Inhibition of Nrf2 by the specific inhibitor brusatol (40 nM)^15^ for 4 h significantly decreased HBXIP promoter activity and HBXIP protein levels in MCF-7 cells and MDA-MB-436 cells (Supplementary Fig. 1E). These results suggest that the transcription factor Nrf2 contributes to *HBXIP* gene expression in breast cancer cells following detachment.

### Functional validation of putative Nrf2 binding sites in the *HBXIP* promoter

**Fig. 3:**
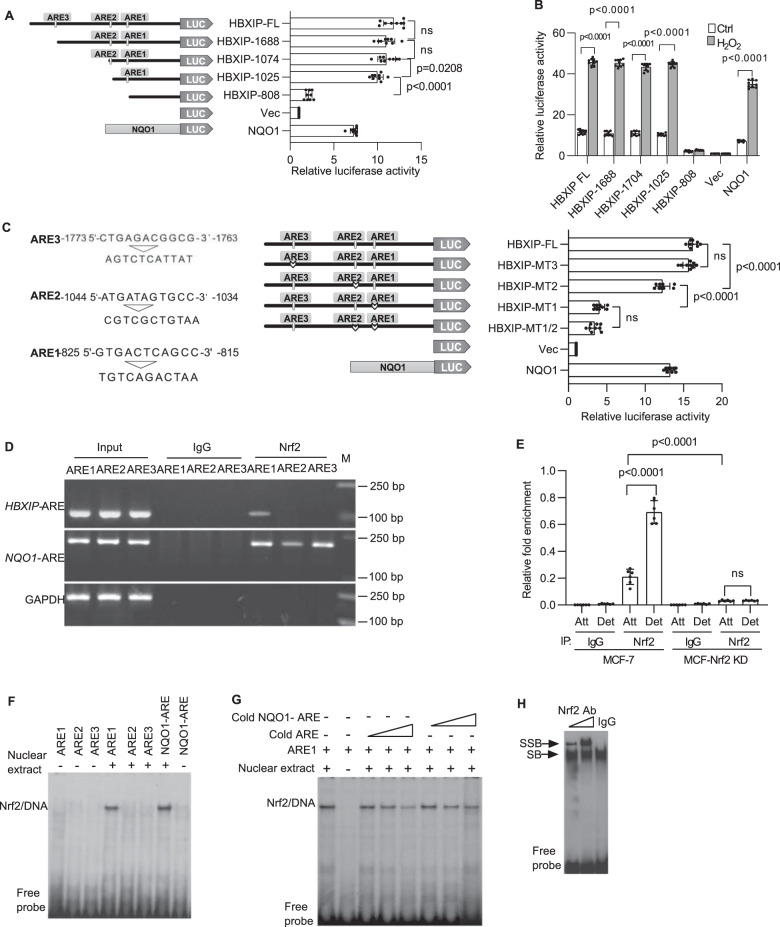
Functional validation of putative Nrf2 binding sites in the *HBXIP* promoter. **A** Systematic representation and strategy for cloning the human *HBXIP* gene promoter into the pGL3-Basic luciferase reporter vector. Three putative AREs (from nt -825 to -815 (ARE1), −1044 to −1034 (ARE2), and −1773 to −1763 (ARE3)) in the *HBXIP* promoter are shown (left panel). *HBXIP* promoter deletion mutants and the *NQO1* promoter were separately cloned into the pGL3-Basic reporter vector and subsequently transfected into MCF-7 cells, and luciferase activity was measured (right panels). Vec, pGL3-Basic vector control. **B** MCF-7 cells transfected with plasmids carrying the full-length *HBXIP* promoter, truncated *HBXIP* promoter deletion mutant constructs, as indicated in **A**, and the *NQO1* promoter was treated with DMSO or 50 μM H_2_O_2_ for 24 h, and then luciferase activity was measured. **C** The putative AREs were mutated as indicated (left panel). Plasmids carrying the full-length *HBXIP* promoter and the indicated mutated *HBXIP* promoters were transfected into MCF-7 cells, and luciferase activity was assessed. The human *NQO1*-ARE luciferase reporter plasmid was transfected into MCF-7 cells as a positive control. Vec, vector control. **D** ChIP assay. MCF-7 cells detached for 8 h were fixed with formaldehyde and cross-linked, and chromatin was sheared ultrasonically. Chromatin was immunoprecipitated with an anti-Nrf2 rabbit monoclonal antibody (mAb) or rabbit (DA1E) control IgG mAb. Nrf2 binding to the *HBXIP* promoter was analyzed by PCR with primers (Supplementary Table 2) specific for the ARE1, ARE2, and ARE3 regions in the *HBXIP* promoter. GAPDH primers were used as a negative control. The three ARE regions in the *HBXIP* promoter were amplified from 5 μl of purified soluble chromatin before immunoprecipitation for use as input DNA. The binding of Nrf2 to the *NQO1*-*ARE* promoter was used as a positive control. **E** ChIP and qRT-PCR. Chromatin was immunoprecipitated from attached and detached MCF-7 cells or stable MCF-Nrf2 KD cells as described in **D**. The binding of Nrf2 to the ARE1 region of the *HBXIP* promoter was measured using qRT-PCR. The amounts of immunoprecipitated DNA were normalized to those of the inputs, and the values were plotted. **F** *HBXIP* AREs (Supplementary Table 5) were end-labeled with [γ-^32^P]ATP and T4 kinase. Labeled DNA (100,000 cpm) was incubated with 10 μg of MCF-7 nuclear extract in binding buffer. The reaction mixtures were separated on a polyacrylamide gel and autoradiographed. DNA-protein complexes containing Nrf2 are indicated. **G** The nuclear proteins binding to ARE1 were competed with increasing doses of cold ARE1 or cold *NQO1*-ARE. **H** The nuclear protein/ARE1 complexes were supershifted with IgG and Nrf2 antibodies. SB shifted band, SSB supershifted band. The error bars indicate the ±SD values as assessed by Student’s *t* test. All experiments were performed at least three times.

The oxidative defense factor Nrf2, a critical trans-acting transcriptional activator that heterodimerizes with small Maf proteins, activates cytoprotective gene transcription by binding to a cis-acting element called the antioxidant responsive element (ARE) in promoter sequences^16^. Accordingly, the *HBXIP* promoter sequence was analyzed using the Cistrome Analysis Pipeline (http://cistrome.org/)^17^ to explore putative cis-acting AREs. Three putative AREs located in the promoter region between nucleotide positions −825 and −815 (ARE1), −1044 and −1034 (ARE2), and −1773 and −1763 (ARE3) from the transcription start site (TSS) were identified in the *HBXIP* promoter (Supplementary Fig. 1F). Accordingly, the full-length *HBXIP* promoter and multiple *HBXIP* promoters with the indicated deletion mutations were cloned into the pGL3-Basic vector to investigate the functionality of these putative AREs (Fig. 3A, left panel). The promoter of human NADH quinone oxidoreductase (*NQO1*)^9^, which contains an ARE, was cloned into the same vector and used as a positive Nrf2-activatable control. The constructed promoter-containing luciferase reporter plasmids were transfected into MCF-7 cells, and the effective ARE was identified according to the luciferase activity. As shown in Fig. 3A (right panel), luciferase reporter activity was prominently invoked if ARE1 was contained in the promoter. Moreover, upon treatment of the transfected MCF-7 cells described above with H_2_O_2_, the full-length *HBXIP* promoter-Luc construct and the deletion mutant *HBXIP* promoter-Luc constructs containing ARE1, as well as the positive control *NQO1-ARE* promoter-Luc construct, showed obviously enhanced luciferase activity (Fig. 3B). Next, three mutated *HBXIP* promoter sequences (Fig. 3C, left panel) constructed using site-directed mutagenesis were cloned separately into the pGL3-Basic vector (Fig. 3C, middle panel), and luciferase activity was evaluated in MCF-7 cells to further verify the functionality of ARE1-ARE3. When the *HBXIP*-MT1 group was compared with the other indicated groups, only the loss-of-function mutation in the ARE1 element resulted in a significant reduction in *HBXIP* promoter-driven luciferase activity (Fig. 3C, right panel). Therefore, ARE1 is the effective cis-acting element for Nrf2-mediated regulation of *HBXIP* gene expression.

Subsequently, we performed ChIP assays and EMSAs to ascertain whether Nrf2 binds to the *HBXIP* gene promoter. Chromosomal DNA was immunoprecipitated from MCF-7 cells using a specific anti-Nrf2 antibody, and PCR arrays for the ARE1, ARE2, and ARE3 regions were analyzed with the help of the corresponding specific primers (Supplementary Table 2). Consistent with the data shown in Fig. 3A–C, specific amplified PCR products from the ARE1 and NQO1-ARE (positive control)-immunoprecipitated chromatin groups were detected in the ChIP assays, demonstrating that ARE1 is the specific binding site for Nrf2 (Fig. 3D). The specific interaction between Nrf2 and ARE1 was also verified in Her2-positive SK-BR3 and TNBC MDA-MB-436 breast cancer cells (Supplementary Fig. 1G). Next, a ChIP-qPCR assay was employed to compare the binding between Nrf2 and ARE1 in attached and detached MCF-7 cells. As expected, ECM detachment induced an increase in Nrf2 binding to ARE1 in the *HBXIP* promoter, and this increase was abolished under Nrf2 knockdown conditions (Fig. 3E). To further support the specific interaction between Nrf2 and ARE1 in the *HBXIP* promoter, we performed EMSA with probes derived from the corresponding putative ARE core regions (Supplementary Table 5), and we indeed observed the DNA-Nrf2 complex only in the ARE1 group (Fig. 3F). Mutating the core ARE1 sequence (A and T to G; G and C to A) completely inhibited the interaction between Nrf2 and ARE1 (Supplementary Fig. 1H). Moreover, the interaction between Nrf2 and the ARE1 probe was competitively inhibited by cold ARE1 and cold NQO1-ARE probes (Fig. 3G). The supershift of the ARE1-Nrf2 complex induced by the anti-Nrf2 antibody further verified the binding specificity (Fig. 3H). These results indicate that Nrf2 binds to the promoter ARE1 site to activate *HBXIP* gene expression and *HBXIP* is a target gene of Nrf2.

### The HBXIP/Nrf2 feedback loop regulates redox homeostasis in breast cancer cells following detachment

**Fig. 4:**
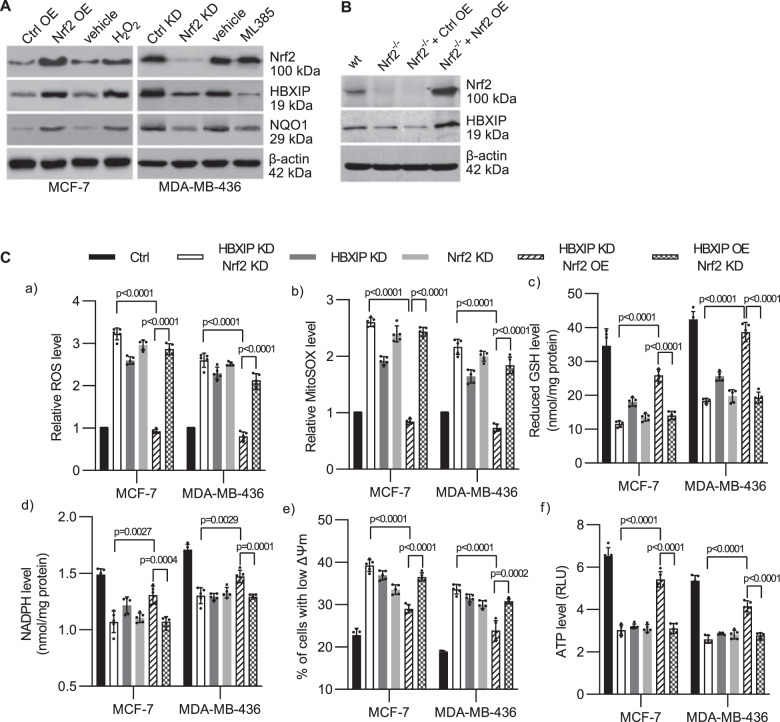
The HBXIP/Nrf2 feedback loop regulates redox homeostasis in breast cancer cells following detachment. **A** MCF-7 cells were infected with pLVSIN-Nrf2 lentivirus or treated with 50 μM H_2_O_2_. MDA-MB-436 cells were infected with pLKO-shHBXIP lentivirus or treated with 5 μM ML385. The indicated treated cells were fractionated, and the expression of HBXIP and NQO1 was measured. **B** Wild-type MEFs and Nrf2^−/−^ MEFs infected with pLVSIN-Nrf2 lentivirus were fractionated, and the levels of Nrf2 and HBXIP were analyzed via western blotting. **C** Stable MCF-7 and MDA-MB-436 cells as indicated were cultured in suspension for 48 h and (a) stained with CM-H_2_DCFDA and subjected to flow cytometry to determine the intracellular ROS level; (b) stained with MitoSOX Red, and the ROS levels were determined; (c) used to measure the reduced GSH levels; (d) used to measure NADPH levels; (e) used for assessment of mitochondrial membrane potential using a JC1 assay; and (f) used for ATP measurement using an ATP determination kit. The error bars indicate the ±SD values as assessed by Student’s *t* test. All the experiments were performed at least three times.

We further validated the effect of Nrf2 on HBXIP protein expression. Upregulating the expression of Nrf2 in MCF-7 cells (which exhibit relatively low HBXIP and Nrf2 expression compared with MDA-MB-436 cells^9^ (Supplementary Fig. 1A)) by infection with LV-Nrf2 lentivirus or treating cells with H_2_O_2_ to activate Nrf2 led to significant upregulation of HBXIP and NQO1 protein expression (Fig. 4A, left panel). Conversely, downregulating Nrf2 by infecting MDA-MB-436 cells (which exhibit relatively high HBXIP and Nrf2 expression^9^ (Supplementary Fig. 1A)) with an LK-shNrf2 lentivirus or inactivating Nrf2 by incubating cells with the Nrf2-specific inhibitor ML385, which blocks Nrf2 heterodimerization with small Maf proteins, potently downregulated HBXIP and NQO1 protein expression (Fig. 4A, right panel). Additionally, extremely low HBXIP expression was found in Nrf2^−/−^ MEFs, and reinstating Nrf2 expression in Nrf2^-/-^ MEFs upregulated HBXIP expression (Fig. 4B). These data suggest that HBXIP expression is regulated by Nrf2. Intriguingly, our previous study showed that HBXIP competitively interacts with Keap1 to activate Nrf2 and its downstream ARE genes in breast cancer^9^. Here, Nrf2 directly bound to the *HBXIP* promoter ARE and activated HBXIP expression. HBXIP and Nrf2 form a positive feedback loop, and the time-course increase in the expression of both HBXIP and Nrf2 is almost consistent in MCF-7 and MDA-MB-231 cells following ECM detachment (Supplementary Fig. 1I). Thus, the HBXIP/Nrf2 axis may induce anoikis resistance in breast cancer.

ECM detachment can lead to harmful metabolic changes, including increased ROS accumulation, attenuated pentose phosphate pathway (PPP) flux and decreased ATP levels^18^. We investigated whether the HBXIP/Nrf2 feedback loop affects redox homeostasis by employing indicated stable breast cancer cells following ECM detachment. HBXIP and Nrf2 expression^9^ in the indicated stable cell lines is shown in Supplementary Fig. 1J. As shown in Fig. 4C and Supplementary Fig. 2A, HBXIP or Nrf2 knockdown (or overexpression) and simultaneous HBXIP and Nrf2 knockdown (or overexpression) significantly upregulated (or downregulated) intracellular ROS levels and mtROS levels, enhanced (or attenuated) mitochondrial permeabilization, decreased (or increased) levels of the two most abundant antioxidants GSH and NADPH, and attenuated (or enhanced) ATP production in breast cancer cells following ECM detachment (Fig. 4C and Supplementary Fig. 2A). Single knockdown (or overexpression) of HBXIP (or Nrf2) had effects similar to those of double knockdown (or overexpression) of the HBXIP/Nrf2 axis (Fig. 4C and Supplementary Fig. 2A). Moreover, compared with HBXIP KD/Nrf2 OE cells, HBXIP OE/Nrf2 KD had limited effects on rescuing the oxidate situation, PPP flux, mitochondrial permeabilization and ATP production relative to HBXIP/Nrf2 KD cells following ECM detachment (Fig. 4C and Supplementary Fig. 2A). These results demonstrate that HBXIP alone has little ability to modulate ROS, PPP flux, and ATP production unless Nrf2 is co-expressed, which is consistent with our previous study showing that HBXIP modulates ROS in breast cancer cells by competing with Nrf2 for binding with KEAP1 and promoting Nrf2 accumulation and activation^9^. The positive HBXIP/Nrf2 feedback loop might reinforce the antioxidant abilities of ECM-detached breast cancer cells to induce anoikis resistance.

### Prdx1/JNK signaling is required for HBXIP/Nrf2 feedback loop-induced anoikis resistance

**Fig. 5:**
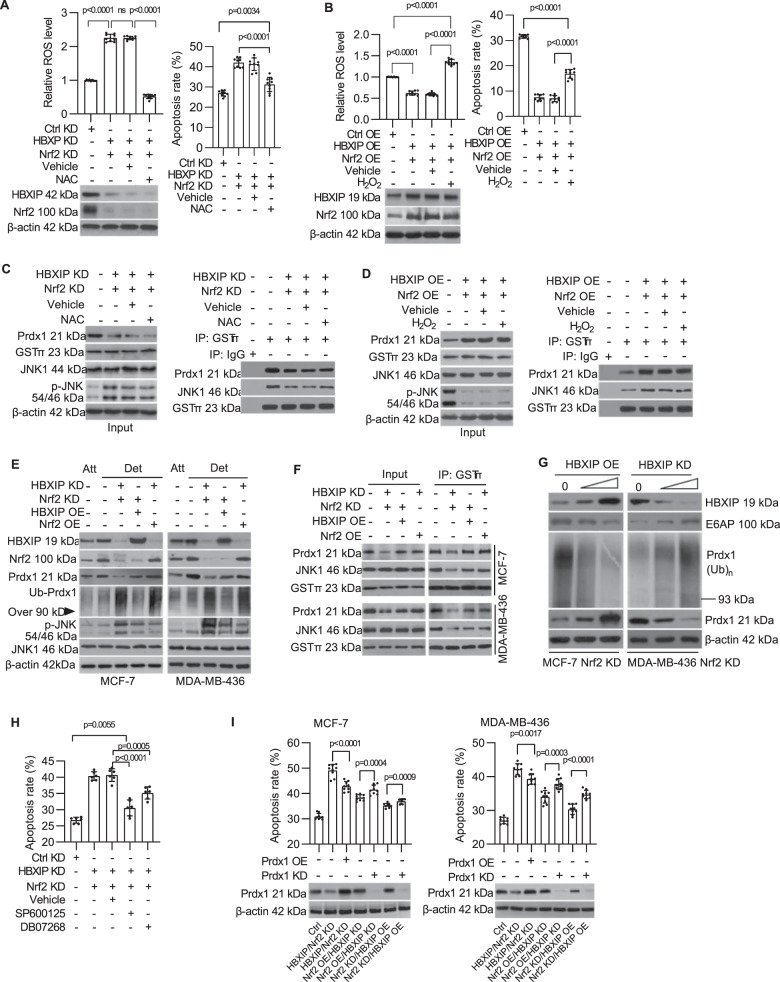
Prdx1/JNK signaling is required for HBXIP/Nrf2 feedback loop-induced anoikis resistance. **A** MDA-MB-436 cells with stable knockdown of the HBXIP/Nrf2 axis were cultured in suspension for 48 h with or without NAC, stained with CM-H_2_DCFDA or Annexin V-FITC and subjected to flow cytometry to determine the intracellular ROS level (left panel) and apoptosis rate (right panel). The immunoblot shows the expression of HBXIP and Nrf2 in the indicated cells. **B** MCF-7 cells stably overexpressing HBXIP/Nrf2 axis were cultured in suspension for 48 h with or without H_2_O_2_, stained with CM-H_2_DCFDA or Annexin V-FITC and subjected to flow cytometry to determine the intracellular ROS level (left panel) or to an Annexin V assay (right panel). The immunoblot shows the expression of HBXIP and Nrf2 in the indicated cells. **C** The indicated cells were cultured and treated as indicated in **A** and subjected to immunoblotting to assess the levels of Prdx1, GSTπ, JNK1, and phosphorylated JNK1 (left panel). Aliquots of cellular protein extracts were subjected to immunoprecipitation with an anti-GSTπ antibody. Goat IgG was used as a negative control. The coimmunoprecipitants were probed for the presence of JNK1 or Prdx1 by western blotting. Western blotting for GSTπ was performed as a loading control (right panel). **D** The indicated cells were cultured and treated as indicated in **B** and subjected to immunoblotting to assess the levels of Prdx1, GSTπ, JNK1, and phosphorylated JNK1 (left panel). Aliquots of cellular protein extracts were subjected to immunoprecipitation with an anti-GSTπ antibody. Goat IgG was used as a negative control. The coimmunoprecipitants were probed for the presence of JNK1 or Prdx1 via western blotting. Western blotting for GSTπ was performed as a loading control (right panel). **E** The indicated stable cell lines cultured in suspension for 8 h were subjected to immunoblotting to assess the HBXIP, Prdx1, ubiquitinated Prdx1, phosphorylated JNK, and JNK1 levels. **F** Aliquots of cellular protein extracts from the indicated stable cell lines were subjected to immunoprecipitation with an anti-GSTπ antibody. The coimmunoprecipitants were probed for the presence of JNK1 or Prdx1 via western blotting. Western blotting for GSTπ was performed as a loading control. **G** Nrf2-deficient MCF-7 and MDA-MB-436 cells infected separately with increasing doses of pLVSIN-HBXIP lentivirus or pLKO-shHBXIP lentivirus were cultured in suspension for 48 h, and the levels of E6AP, Prdx1, and ubiquitinated Prdx1 were measured via western blotting. **H** After treatment of HBXIP/Nrf2 double knockdown MDA-MB-436 cells with 40 nM SP600125 or 9 nM DB07268, which are JNK inhibitors, changes in the rate of cellular anoikis were detected. **I** The indicated stable cell lines with Prdx1 overexpression (or knockdown) induced by infection with recombinant lentivirus, were cultured in suspension, stained with Annexin V-FITC and subjected to flow cytometry to determine the apoptosis rate. The immunoblot shows Prdx1 expression in the indicated cells. The error bars indicate the ±SD values as assessed by Student’s *t* test. All experiments were performed at least three times.

Antioxidant *N*-acetyl-l-cysteine (NAC) could mimic the effect of the HBXIP/Nrf2 axis to eliminate cellular ROS in detached MDA-MB-436 cells. However, the increased apoptosis rate induced by blocking the HBXIP/Nrf2 axis was only partially rescued by NAC treatment (Fig. 5A). Additionally, elevating the intracellular ROS level following H_2_O_2_ treatment caused a partial increase in the apoptosis rate of HBXIP/Nrf2-enforced MCF-7 cells (Fig. 5B). These results suggest that maintaining robust intracellular ROS levels is only one of the necessary conditions for breast cancer cell survival following ECM detachment.

Prdx1, a target factor of Nrf2^19^, acts as a molecular chaperone to protect tumor cells from apoptosis by inhibiting JNK1 activation^20^. We investigated whether the Prdx1/JNK1 signaling was involved in the HBXIP/Nrf2 feedback loop-mediated anoikis resistance. Knockdown of the HBXIP/Nrf2 axis significantly downregulated Prdx1 expression and increased the level of proapoptotic phospho-JNK1 (Fig. 5C, left panel). By contrast, overexpressing the HBXIP/Nrf2 axis significantly increased Prdx1 expression and decreased the phospho-JNK1 levels in ECM-detached MCF-7 cells (Fig. 5D, left panel). In addition, we found that both HBXIP and Nrf2 have positive effects on Prdx1 protein accumulation in cells following ECM detachment. As shown in Fig. 5E, the Prdx1 protein levels in HBXIP OE/Nrf2 KD cells and HBXIP KD/Nrf2 OE cells were apparently higher than in HBXIP/Nrf2 double KD cells following ECM detachment. Meanwhile, the levels of ubiquitinated Prdx1 in HBXIP KD/Nrf2 OE cells and HBXIP/Nrf2 double KD cells were apparently higher than in HBXIP OE/Nrf2 KD cells (Fig. 5E). Moreover, in ECM-detached Nrf2 knockdown breast cancer cells, the Prdx1 level was positively correlated with the HBXIP level, but the ubiquitinated Prdx1 level showed the opposite trend (Fig. 5G). Collectively, these findings suggest that the role of HBXIP in regulating Prdx1 accumulation is mainly focused on inhibiting Prdx1 ubiquitination and degradation and that Nrf2 promotes Prdx1 expression. Moreover, the JNK phosphorylation level was negatively correlated with the Prdx1 protein level in the indicated ECM-detached stable cell lines shown in Fig. 5E. Use of the antioxidant NAC to neutralize ROS (or H_2_O_2_ treatment) showed little effect on JNK1 phosphorylation or the Prdx1 protein levels in ECM-detached HBXIP/Nrf2-deficient MDA-MB-436 cells (or HBXIP/Nrf2 double OE MCF-7 cells) (Fig. 5C, left panel and Fig. 5D, left panel). This pattern suggests that Prdx1-mediated JNK1 activation is involved in HBXIP/Nrf2 feedback loop-induced anoikis resistance in addition to diminishing ROS levels in breast cancer cells.

Previous research confirmed that Prdx1 interacts with the GSTπ-JNK1 complex and suppresses the release of JNK1 from the complex, which inhibits JNK1 phosphorylation and activation^20^. The co-immunoprecipitation assay showed that Prdx1 downregulation led to dissociation of the Prdx1-GSTπ-JNK1 complex in HBXIP/Nrf2 feedback loop-deficient breast cancer cells (Fig. 5C, right panel and 5F). Treatment with the antioxidant NAC did not rescue Prdx1 expression or prevent the dissociation of JNK1 from the heterotrimeric complexes (Fig. 5C, right panel). Enforced HBXIP/Nrf2 expression (or HBXIP or Nrf2 overexpression in HBXIP/Nrf2 KD cells) upregulated Prdx1 and stabilized the heterotrimeric complexes, and oxidant (H_2_O_2_) treatment did not block the Prdx1-GSTπ-JNK1 interaction (Fig. 5D, right panel and Fig. 5F). We found that HBXIP promotes Prdx1 accumulation in cells by inhibiting E6AP expression, which induced ubiquitin-mediated degradation of Prdx1 (Fig. 5G). Furthermore, inhibiting JNK1 activation with the specific inhibitor SP600125 (or DB07268) promoted the survival of HBXIP/Nrf2-deficient, ECM-detached cells (Fig. 5H). Rescued overexpression of Prdx1 decreased the apoptosis rate of HBXIP/Nrf2 doubles KD breast cancer cells following ECM detachment. Prdx1 knockdown increased the apoptosis rate of HBXIP OE/Nrf2 KD and HBXIP KD/Nrf2 OE breast cancer cells (Fig. 5I). Thus, these results indicate that the reciprocal HBXIP/Nrf2 feedback loop not only prevents unconstrained ROS accumulation but also promotes Prdx1 accumulation and stabilizes the Prdx1-GSTπ-JNK1 complex to reduce JNK1 phosphorylation following ECM detachment.

### The HBXIP/Nrf2 feedback loop induces anoikis resistance and metastasis in breast cancer cells in vivo

**Fig. 6:**
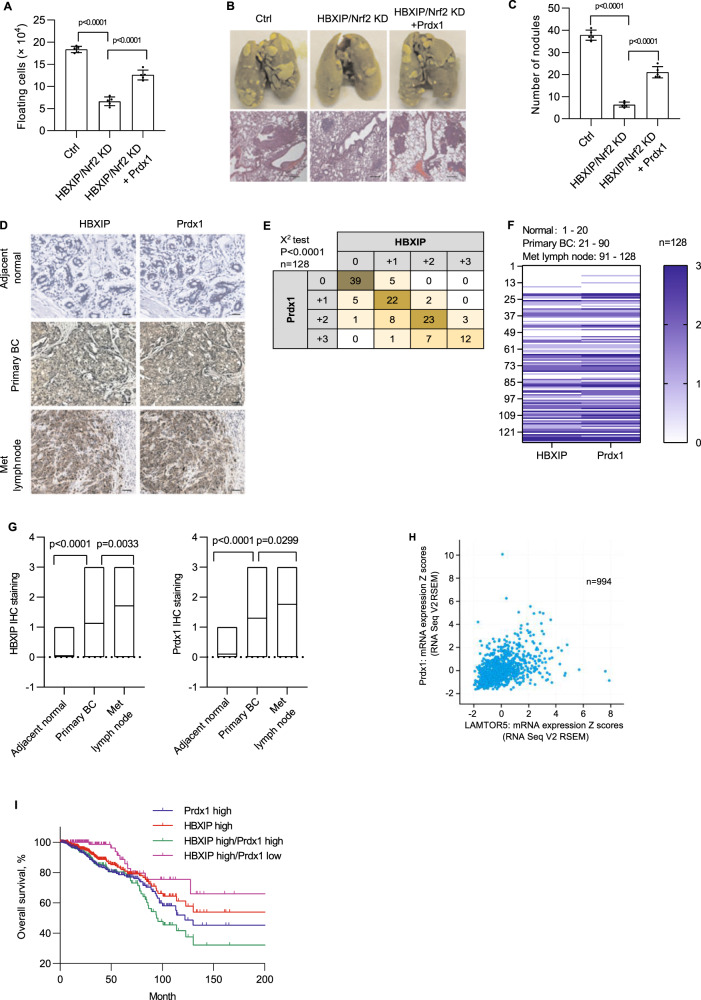
The HBXIP/Nrf2 feedback loop induces metastasis and anoikis resistance in breast cancer cells in vivo. **A** At 4 days after the i.p. injection with stable HBXIP/Nrf2 KD MDA-MB-436 cells and stable Prdx1 OE HBXIP/Nrf2-deficient MDA-MB-436 cells, the viable cells were counted using the trypan blue exclusion assay after removing possible contaminating mouse cells. **B** Representative images of lung tissue and tissue sections from the indicated groups of mice were obtained 3 weeks after the tail vein injection of the indicated stable breast cancer cells. Scale bars, 200 μm. **C** The lung metastatic nodules in the mouse lungs shown in **B** were counted. **D** The expression of the HBXIP and Prdx1 proteins in normal breast tissues, primary breast cancer tissues, and metastatic lymph node tissues were examined using IHC. Scale bars, 50 μm. **E** The association between HBXIP and Prdx1 expression levels in the abovementioned IHC assay was statistically analyzed by *χ*^2^ test. **F** Heatmap of HBXIP and Prdx1 expression in the tumor tissues from each patient referenced in **D**. **G** Correlations between HBXIP and Prdx1 expression in patients with breast cancer are referenced in panel **F**. **H** Expression levels of the *HBXIP* and *Prdx1* mRNAs (*z* scores, RNA Seq V2 RSEM) were analyzed in 994 clinical breast cancer samples (Pearson’s correlation coefficient; *r* = 0.43, Spearman’s correlation coefficient = 0.47). **I** The patients with breast cancer referenced in **H** were filtered into four groups (Prdx1 high = 359, HBXIP high = 354, HBXIP high/Prdx1 high = 215, HBXIP high/Prdx1 low = 139) according to the HBXIP and Prdx1 expression levels and *z* scores. An overall survival curve was generated using the Kaplan–Meier method, and results were statistically compared using a log-rank test. *p* values were determined using Student’s *t* test for the data shown in (**A**, **C**, and **G**) and using a chi-square test for the data in **E**.

To confirm whether the HBXIP/Nrf2 feedback loop-induced in vivo anoikis resistance, we established an ascites tumor model by injecting the indicated breast cancer cells into the peritoneal cavity of Balb/c mice. Four days after the i.p. injection, cell viability assays showed that HBXIP/Nrf2 axis-deficient cells exerted more reduced cell viability than the control group (Fig. 6A). The rescued overexpression of Prdx1 increased the survival rate of HBXIP/Nrf2 axis-deficient cells. To further determine the effect of the HBXIP/Nrf2 feedback loop on metastasis, the indicated breast cancer cells were injected into the tail vein of Balb/c mice. Pathological examination of lung metastasis showed that knockdown of the HBXIP/Nrf2 axis significantly inhibited the lung metastasis of breast cancer cells. The rescued overexpression of Prdx1 in HBXIP/Nrf2 axis-deficient cells restored the metastatic ability of tumor cells (Fig. 6B, C).

**Fig. 7:**
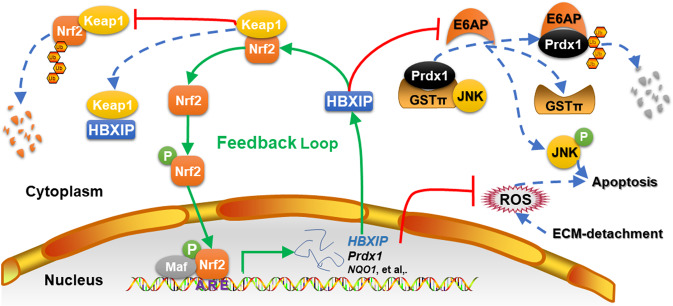
Summary model of the reciprocal HBXIP/Nrf2 feedback loop in breast cancer cells. HBXIP induces anoikis resistance in breast cancer cells by forming a reciprocal positive feedback loop with Nrf2 to maintain redox homeostasis, inhibiting JNK1 activation by preventing Prdx1 ubiquitination.

Because the cellular Prdx1 protein level is also affected by HBXIP in addition to Nrf2 in breast cancer cells (Fig. 5), we tested whether these two proteins are correlated in clinical breast cancer tissues. The expression status of HBXIP and Prdx1 in 128 breast cancer tissues (Supplementary Table 6) was determined using immunohistochemical (IHC) staining. The staining intensity of HBXIP was positively correlated with that of Prdx1 (Fig. 6D, E), and the Prdx1 and HBXIP expression levels were significantly correlated with the status of metastasis in breast cancer tissues (Fig. 6F, G). These findings were further validated clinically by analyzing the cBioPortal cancer genomics database of breast cancer datasets. In total, 994 breast invasive carcinoma patients with complete genomic data (TCGA, PanCancer Atlas, Cell 2018) were collected from cBioPortal for Cancer Genomics database^21,22^. The clinical characteristics of patients are summarized in Supplementary Table 7. The expression data were normalized to *z* scores and high (or low) expression was defined by a positive (or negative) *z*-score value. A markedly positive correlation was observed between HBXIP expression and Prdx1 expression in 994 patients with breast cancer (Fig. 5H). In addition, a significant correlation was found between the HBXIP and Prdx1 protein expression levels (mass spectrometry, CPTAC) in 104 patients (in 994 patients with protein expression data) (Supplementary Fig. 2B, C). Breast carcinoma patients with different risks of cancer progression or death were filtered into four groups (HBIXP high expression group, Prdx1 high expression group, HBXIP/Prdx1 high group, and HBXIP high/Prdx1 low group) based on HBXIP and Prdx1 expression z scores. A log-rank test showed that patients with high HBXIP and Prdx1 co-expression had the worst outcome compared with patients with high expression of either HBXIP or Prdx1 alone (Fig. 6I). The overall survival of the HBXIP high/Prdx1 low group was not significantly different from that of the HBIXP high group (Fig. 6I). We next explored 2463 breast cancer patients filtered by HBXIP expression on Kaplan–Meier Plotter platforms. A log-rank test showed that the recurrence-free survival (RFS) outcome in the HBXIP high/Prdx1 high group was worse than that in the HBXIP high/Prdx1 low group (Supplementary Fig. 2D). This pattern indicates that Prdx1 accumulation mediated by HBXIP contributes to breast cancer development. In summary, the reciprocal HBXIP/Nrf2 feedback loop promotes anoikis resistance in breast cancer cells both in vitro and in vivo by maintaining robust cellular ROS levels and Prdx1 stabilization, which inhibits the release of JNK1 from Prdx1-GSTπ-JNK1 heterotrimeric complexes and subsequently inhibits JNK activation (Fig. 7). Simultaneous inhibition of HBXIP and Nrf2 expression is a possible effective treatment for breast cancer.

## Discussion

Metastatic breast cancer cells must be able to resist anoikis to survive under anchorage-independent conditions when they circulate in the bloodstream. The molecular mechanisms of anoikis resistance in invasive breast cancer cells are complicated. The findings in our present study outline a mechanism by revealing the involvement of a reciprocal feedback loop between HBXIP and Nrf2 in inducing anoikis resistance. Breast cancer cells employ HBXIP, which competes with Nrf2 for binding to the Keap1 protein, to activate the Nrf2-ARE pathway^9^. Interestingly, HBXIP was identified as a target ARE-gene of Nrf2 (Figs. 2–4). Therefore, a reciprocal feedback loop is formed between HBXIP and Nrf2 in breast cancer cells. The HBXIP/Nrf2 axis promotes ECM-detached breast cancer cell survival by reducing cellular ROS accumulation and stabilizing Prdx1, which inhibits the release of JNK1 from Prdx1-GSTπ-JNK heterotrimeric complexes and prevents JNK1 activation (Fig. 7). Therefore, targeting the HBXIP/Nrf2 axis is a promising therapeutic method for breast cancer treatment.

Cancer cell detachment is often accompanied by increased cellular ROS accumulation, which is indispensable for anoikis resistance^23^. Intracellular ROS and ATP are mainly generated by mitochondria, and cell death caused by alterations in mitochondrial metabolism is closely related to loss of the mitochondrial membrane potential^24^. ROS perform various functions following cell detachment, such as activating numerous redox-sensitive TFs (p53 AP-1, HIF-1α, and NF-kB)^25^ and signaling pathways (PI3K/AKT and EGFR)^26,27^. However, uncontrolled excessive ROS accumulation is lethal to cancer cells, and the activity of both JNK and caspases is sensitive to the redox state of the cell^28^. Tumor cells must evolve powerful antioxidant systems to maintain ROS at a robust, subtoxic level for survival. The reciprocal HBXIP/Nrf2 feedback loop present in this study is an important means to accomplish this effect. On the one hand, activated Nrf2 activates numerous antioxidative and cellular protection genes, such as *NQO1, GCLC, HO-1*^16^, and, notably, *HBXIP* (Figs. 2 and 3). On the other hand, upregulated HBXIP not only competes with Nrf2 for Keap1 binding and promotes Nrf2-ARE activation^9^ but also functions as a transcriptional coactivator to promote a malignant phenotype^6,8^. Additionally, the enforced HBXIP/Nrf2 feedback loop promotes the survival of ECM-detached breast cancer cells through enhanced antioxidant-scavenging ability by maintaining the mitochondrial membrane potential and ATP generation to accumulate GSH and NAPDH (Fig. 4). Consistent with our study, antioxidants promote anchorage-independent survival of breast cancer cells by restoring ATP generation^29^. Recent studies have shown that diminishing ROS by obstructing the flux of glycolytic carbon into mitochondrial oxidation by upregulated pyruvate dehydrogenase kinase 4 enhances cell survival during ECM detachment^30^. Because maintaining a robust ROS level is important for tumor cell survival and malignant phenotypic progression, we hypothesize that breast cancer cells have multiple mechanisms, including the HBXIP/Nrf2 feedback pathway, to resist excessive ROS production during ECM detachment. Moreover, hyperactivated HBXIP and Nrf2 have been found in chemo-/radiotherapy resistant breast cancer^11,31^. One possible reason for the development of chemo-/radiotherapy resistance in breast cancer is that the ROS induced by chemo-/radiotherapy can activate positive HBXIP/Nrf2 feedback (Fig. 5 and Supplementary Fig. 1K) and reinforce their respective tumor-promoting actions^32,33^. Therefore, simultaneous inhibition of HBXIP/Nrf2 is a promising therapeutic approach to enhance the efficacy of breast cancer chemo-/radiotherapy.

The antioxidant ROS scavenger Prdx1 is a member of the redox-regulating peroxiredoxin family. Abnormal Prdx1 expression has been observed in several human cancers, including breast, colon, esophageal, ovarian, oral, lung, and prostate cancers^34–38^. *Prdx1* is a cis-acting ARE-containing target gene of Nrf2^19^ that plays critical role in promoting cancer growth via its diverse activities in metabolizing ROS and serving as a molecular chaperone^39–41^. The aggressive survival phenotype-promoting function of Prdx1 is traditionally attributed to its antioxidant activity. However, emerging studies have revealed that the cancer-promoting actions of Prdx1 can be explained by its physical interaction with different vital regulatory effectors of proliferation and apoptosis and regulation of their activities^41,42^. For instance, Prdx1 interacts with the Src homology-3 domain of c-Abl^43^, the Myc Box II domain of c-Myc^44^, the macrophage inhibiting factor MIF^45^, and the C2 domain of PTEN^36^. These newly explored roles of Prdx1 are independent of its peroxide-detoxifying function, consistent with our findings that Prdx1 suppresses JNK1 activation by forming Prdx1-GSTπ-JNK1 heterotrimeric complexes during ECM detachment of tumor cells. The Prdx1 C52S mutant, which has no antioxidant activity, produced essentially the same results as wild-type Prdx1 on apoptosis inhibition (Supplementary Fig. 3). Our results are consistent with those of a previous study in lung cancer showing that the antioxidant activity and the apoptosis prevention function of JNK inhibition are not associated^20^. Additionally, although Nrf2 is as an essential TF for *Prdx1* gene expression^19^, our clinical IHC staining analysis and clinical data analysis showed a marked correlation not between Nrf2 and Prdx1 but between HBXIP and Prdx1. High co-expression of HBXIP/Prdx1 predicts a worse survival outcome than high expression of either alone (Fig. 6). Our findings are consistent with those of a previous study on non-small cell lung cancer (NSCLC) indicating that the expression of Prdx1 was not statistically significantly correlated with the expression of Nrf2^35^. Elevated expression of Prdx1, but not Nrf2, is an independent prognostic factor for NSCLC^35^. In addition, the accumulation of the Prdx1 mRNA and protein was reported to be diminished but not completely abolished in Nrf2 knockout MEFs^19^. These results indicate that the mechanism regulating Prdx1 accumulation in various cancers^34–38^ is very complex. In addition to Nrf2, other positive or negative regulatory mechanisms may cooperate to induce the expression of Prdx1 or inhibit its degradation. Our findings in this study provide a possible approach to diminish the elevated expression of Prdx1: inhibiting HBXIP to enhance ubiquitin-dependent degradation of Prdx1.

In conclusion, our present findings present a significant regulatory mechanism by which the reciprocal HBXIP/Nrf2 feedback loop induces anoikis resistance by maintaining robust cellular ROS levels and stabilizing Prdx1 to inhibit the release of JNK1 from Prdx1-GSTπ-JNK heterotrimeric complexes (Fig. 7). These findings offer a new perspective on breast cancer treatment by targeting the HBXIP/Nrf2 axis.

## Methods

### Cell culture, transfection and generation of stable cell lines

The cell lines used in this study and the corresponding culture methods are summarized in Supplementary table 3. *Nrf2*^−/−^ MEF lines^46^ were obtained from Tohoku University (Japan). All plasmids were transfected into indicated subconfluent cells using Lipofectamine 2000 (Invitrogen, Thermo Fisher Scientific) according to the product manual and analyzed 72 h after transfection. Gene-specific or -nonspecific control siRNA duplexes were synthesized by RiboBio (Guangzhou, China) and transfected into cells using the RNAiMAX transfection reagent (Invitrogen, Thermo Fisher Scientific) according to the manufacturer’s handbook. All oligonucleotide sequences used in the present study are provided in Supplementary Table 2.

### Plasmids

The plasmids used in this study are listed in Supplementary Table 1. They were generated using conventional restriction enzyme-based cloning technology. The vectors pCMV-tag2B, psPAX2, pMD2.G, pLKO.1, pLVSIN-CMV neo, pGL3-Basic, and pRL-TK; expression constructs pCMV-HBXIP and pCMV-Nrf2^9^; and lentiviral vectors pLKO-HBXIP (puro^r^)^9^ and pLVSIN-HBXIP (neo^r^)^9^ were maintained in our laboratory. The plasmids containing cDNAs for *SATB1* (BamHI and HindIII, GenBank accession number: M97287.1), *Sox2* (BamHI and HindIII, GenBank accession number: NM_003106.4), *Prdx1* (BamHIand HindIII, GenBank accession number: NM_181696), and *Nrf2* (cloned from pCMV-Nrf2) were generated using regular PCR and separately inserted into pCMV-tag 2B, pLVSIN-CMV pur and pLVSIN-CMV hyg plasmids using the indicated restriction enzymes. The 5’-flanking region (from nt −2156 to +357, +1 TSS) of the *HBXIP* gene was amplified via PCR from the genomic DNA of MCF-7 cells using specific primers and inserted into the ACC65I and MluI sites upstream of the luciferase gene in the pGL3-Basic vector. The resulting plasmid was sequenced and named pGL-HBXIP FL. Several 5′ deletions (−1688/+357, −1074/+357, −1025/+357, and −808/+357) in the *HBXIP* promoter were generated by PCR from pGL-HBXIP FL using specific primers, inserted into the pGL3-Basic vector, and named pGL-HBXIP 1688, pGL-HBXIP1074, and pGL-HBXIP 808, respectively. Constructs containing the mutant HBXIP promoter, which were named HBXIP-MT1, MT2, MT3, and MT1/2, carried substituted nucleotides within the putative AREs and were generated using the GeneArt™ Site-Directed Mutagenesis System (Invitrogen, Thermo Fisher Scientific). The human *NQO1* gene promoter containing an ARE was generated by PCR from hNQO1-ARE-luciferase reporter plasmids, as described in our previous study^9^, and inserted into the NheI and SmaI sites of pGL3-basic plasmid to be used as a positive control. All plasmid constructs were verified by restriction digestion and/or DNA sequencing. The primers used in the present study are listed in Supplementary Table 2.

### Lentivirus production and generation of stable cell lines

A detailed description of the procedures used for lentivirus production and infection is provided in our previous study^9^. Briefly, pLKO-shNrf2 was constructed by cloning *Nrf2* shRNA (5′-CCGGTCCTACTGTGATGTGAAATCTCGAGATTTCACATCACAGTAGGATTTTTG-3′)^47^ into the pLKO.1 vector. The lentivirus LK-shNrf2 carrying *Nrf2* shRNA was generated and harvested 72 h after co-transfection of 293T cells with the lentiviral vectors psPAX2 and pMD2.G. For overexpression, the lentiviral expression vectors pLVSIN-Nrf2 (puro^r^) and pLVSIN-Prdx1 (Hyg^r^) containing the respective coding sequences were used for transfection, and the recombinant viruses LV-Nrf2 and LV-Prdx1 were produced in 293T cells using the Lenti-X ^TM^ HTX Packaging System (CLONTECH Laboratories, Inc.). Lentiviruses were harvested after 72 h.

Cells (50–60% confluence) cultured in serum-free medium containing 8 µg/mL polybrene (Sigma) were infected with the appropriately constructed lentiviruses to generate stable cell lines. Three days after the infection, cells were selected by treatment with either 4 μg/mL puromycin, 800 μg/mL G418, 200 μg/mL hygromycin B, or with a combination of antibiotics for 2 weeks. The qualified stable single-cell clones were screened using the limited dilution method and verified by western blotting.

### RNA extraction and qRT-PCR

Total RNA was extracted from the cells indicated in Fig. 1 using TRIzol reagent (Invitrogen, Thermo Fisher Scientific). First-strand cDNAs were synthesized using PrimeScript reverse transcriptase (TaKaRa) and oligo-(dT) according to the manufacturer’s instructions. Quantitative real-time PCR was performed on an iCycler iQ5 Real Time PCR (Bio-Rad Laboratories, Hercules, CA) machine using iQ SYBR Green Supermix (Bio-Rad Laboratories). All qRT-PCR assays were performed in triplicate in at least three independent experiments. Relative expression levels of each target gene were normalized to the *GAPDH* level. The primers used in the present study are listed in Supplementary Table 2.

### Induction of anoikis

The appropriate cells were re-suspended in complete medium with or without 50 μM H_2_O_2_ (or 5 mM NAC or 5 μM ML385) and cultured on plates coated with poly-HEMA (Sigma), as described in a previous report^29^, to induce anoikis. Particularly, sodium pyruvate-free medium was used for H_2_O_2_ treatment. At the indicated time points, apoptotic cell death induced by detachment was analyzed using an Annexin V-FITC apoptosis detection kit (KeyGEN Biotech) and flow cytometry according to the manufacturer’s protocol.

To maintain a constant H_2_O_2_ concentration in the medium (sodium pyruvate free, Hyclone, Cat# SH30022), the H_2_O_2_ treatments were performed using steady-state titration^48^ according to a previous report^49^. Briefly, the H_2_O_2_ steady-state was achieved by simultaneously adding an initial 50 μM dose of H_2_O_2_ and an adequate amount of glucose oxidase, which was added to compensate for the rapid consumption of the initial H_2_O_2_ and to keep the H_2_O_2_ concentration constant during the entire assay. The H_2_O_2_ concentration in the medium was checked periodically by removing aliquots of the medium and measuring the oxygen released after catalase addition using an oxygen electrode.

### Soft agar colony formation assay

Cells (1 × 10^4^) were suspended in a complete L-15 medium containing 0.35% agar and plated onto six-well plates coated with a 0.6% agar layer. The cells were cultured for 2 weeks at 37 °C and stained with crystal violet. Images were captured and analyzed with ImageJ software (NIH Image, Bethesda, MD).

### Transcription factor activity profiling

MCF-7 cells were cultured under attached or detached conditions for 24 h, and then nuclear proteins were extracted. The activities of 96 transcription factors were determined using TF Activation Profiling Plate Array II with Nuclear Extraction Kit (Signosis) according to the product manual.

### Promoter reporter assay and ChIP-qRT-PCR assay

For the *HBXIP* promoter-reporter assay, the pCMV-SATB1, pCMV-Sox2, or pCMV-Nrf2 constructs were co-transfected separately with the pGL-HBXIP FL promoter construct and pRL-TK normalization construct into MCF-7 cells using Lipofectamine 2000. At 72 h after transfection, the *HBXIP* promoter activity was tested using the dual-luciferase reporter assay (Promega) according to the manufacturer’s instruction.

The ChIP assay was conducted using a QuikChIP ChIP Kit (Novus Biologicals) according to the manufacturer’s instructions. Briefly, the MCF-7 cells were cultured on poly-HEMA-coated plates for 24 h and then fixed with 1% formaldehyde for 15 min. The cells were lysed and the chromatin sonicated (200–1000 bp fragments). The sheared chromatin was immunoprecipitated with an anti-Nrf2 (1:200 dilution, Cell Signaling Technology, Danvers, MA, USA) or control IgG antibody (1:200 dilution, Cell Signaling Technology, Danvers, MA, USA). After removing the protein, the chromatin was subjected to PCR amplification to detect the putative ARE regions of the *HBXIP* promoter bound to Nrf2. *GAPDH* PCR was performed as an internal control, and *NQO1-ARE* PCR was set as a positive control. PCR products were separated on a 2% agarose gel. The relative binding of Nrf2 to ARE1 in attached and detached MCF-7 and stable Nrf2 knockdown MCF-shNrf2 cells was also compared using ChIP-qRT-PCR on an iCycler iQ5 Real Time PCR machine (Bio-Rad Laboratories) using iQ SYBR Green Supermix (Bio-Rad Laboratories) to verify the specific binding of Nrf2 to ARE1 in the *HBXIP* promoter. All primers used in the present study are listed in Supplementary Table 2.

### Electrophoretic mobility shift assay

DNA fragments derived from the putative ARE1, ARE2 and ARE3 core regions in the *HBXIP* promoter were synthesized and 5′-end-labeled with [γ-^32^P]ATP and T4 polynucleotide kinase. The probe sequences are listed in Supplementary Table 2. Labeled AREs (100,000 cpm) were incubated with 10 μg of the MDA-MB-436 nuclear extract in the absence or presence of a 1000-fold molar excess cold ARE1 or cold NQO1-ARE, and a band shift assay was performed using a previously described approach^50^. Based on the experiment, the gel shift nuclear extract mixture was also incubated with 2 μg of control IgG or Nrf2 antibody (Cell Signaling Technology) at 4 °C for 2 h to perform a supershift assay. The mixtures were separated on a 4% polyacrylamide gel and autoradiographed. Probe sequences used in the present study are listed in Supplementary Table 5.

### Transient transfection and luciferase assay

In related experiments, subconfluent transfected or drug-treated cells grown in 24-well plates were co-transfected with the indicated pGL-*HBXIP* promoter or *NQO1-ARE* promoter constructs and pRL-TK normalization construct using Lipofectamine 2000 (Invitrogen). After 24 h, the cells were harvested and lysed, and the luciferase activity was measured using the dual-luciferase assay system (Promega). The relative luciferase activity was calculated and plotted after normalizing the values to Renilla luciferase activity.

### Annexin V-FITC-PI apoptosis analysis

Apoptotic cells were quantified using an Annexin V-FITC-PI apoptosis detection kit (Vazyme Biotech). Briefly, cells pre-treated with the appropriate molecules were resuspended in 500 μl of binding buffer. Afterwards, 5 μl of PI were added and evenly blended after 5 μl of Annexin V-FITC were added. The mixture was reacted for 15 min at room temperature in the dark. Apoptotic cells were detected using flow cytometry (Ex = 488 nm; Em = 530 nm).

### ROS quantification assay

The intracellular ROS levels were measured via flow cytometry using 2,7-dichlorofluorescein diacetate (DCFH-DA) (Nanjing Jiancheng Bioengineering Institute) as a probe. Mitochondrial ROS production was detected using a MitoSOX Red assay (Thermo). Transfected or drug-treated cells in 24-well plates were washed twice with PBS and then incubated with 10 μM DCFH-DA at 37 °C for 30 m or with 5 μM MitoSOX reagent for 10 m in the dark according to the manufacturer’s instructions. The cells were harvested, and the intracellular ROS levels were measured using flow cytometry.

### NADPH, GSH, and ATP assays

The intracellular levels of NADPH were measured using a Coenzyme II(NADP/NADPH) content test kit (Nanjing Jiancheng Bioengineering Institute) according to the manufacturer’s instructions. The intracellular levels of reduced GSH were measured using a Reduced Glutathione (GSH) Assay Kit (Nanjing Jiancheng Bioengineering Institute) according to the manufacturer’s instructions. The intracellular level of ATP was measured using an ATP assay kit (Nanjing Jiancheng Bioengineering Institute). All the measurements were normalized to the protein content of the cells.

### Mitochondrial membrane potential analysis

The mitochondrial membrane potential was measured using a mitochondrial membrane potential detection kit (JC-1 method) (Nanjing Jiancheng Bioengineering Institute) according to the manufacturer’s instructions. Multivariate data analysis was performed using FlowJo software.

### Preparation of cell extracts and immunoblotting

Whole-cell extracts were acquired using a Mammalian Total Protein Extraction Kit (Transgen). Western blot analyses were performed as described previously^9^. All antibodies used in the present study are listed in Supplementary Table 1. We confirmed all blots derive from the same experiment and were processed in parallel. The uncropped scans of the important blots were presented in Supplementary Information.

### Immunohistochemical staining

One hundred twenty-eight paraffin-embedded breast tumor tissues, including 70 malignant carcinomas, 38 metastatic carcinomas, and 20 adjacent normal tissues, were collected from patients undergoing breast cancer resection at the Fourth Hospital of Hebei Medical University (Shijiazhuang, China). The patients’ clinicopathological information is presented in Supplementary Table 6. Two serial sections from each patient were used for IHC staining as described in a previous report^9^. Positive staining was identified by an experienced pathologist using IHC signal intensity scored as 0, +1, +2, and +3.

### TCGA database analysis

Molecular and clinicopathological information of patients with invasive breast cancer was retrieved from the cBioPortal tool (www.cbioportal.org) and comprised 994 eligible samples^51^ (“Breast Invasive Carcinoma (TCGA, PanCancer Atlas)” dataset) with CAN and expression data^22^. Kaplan–Meier survival analysis was performed to estimate the survival distributions, and a log-rank test was performed to assess the statistical significance of differences between the groups.

### In vivo cell survival and metastasis assays

For the analysis of cell survival in the mouse peritoneal cavity, each of the indicated stable cell lines (5 × 10^6^ per mouse) were i.p. injected into female Balb/c mice (6 weeks old, 5 mice per group). Four days after the injection, ascites was drawn from the peritoneal cavity and washed with culture medium. The possible mouse cell contamination in ascites was removed using a mouse cell depletion kit (Miltenyi Biotec) according to the manufacturer’s instructions. Tumor cell viability was determined using the trypan blue dye exclusion method.

For in vivo metastasis assays, each of the indicated stable breast cancer cell lines was harvested in 0.1 mL of PBS and injected into the lateral tail vein of female Balb/c mice (6 weeks old, 5 mice per group, 5 × 10^5^ cells per mouse) housed under SPF conditions. The mice were euthanatized 3 weeks after the injection, and the lungs were resected and fixed with Bouin’s fixative solution (Solarbio). The lungs were imaged, and the nodules were counted using a dissecting microscope at 4× magnification. The fixed lung tissues were then embedded in paraffin, sectioned, and stained with a hematoxylin and eosin staining kit (Solarbio). All animal studies were complied with relevant ethical regulations for animal testing and research, and all experiments conducted in the study received approval from the Institutional Animal Care and Use Committee of the Hebei University of Science and Technology.

### Statistical analysis

A two-tailed Student’s *t* test was used to compare data between two groups. Data are presented as mean ± SD values. Kaplan–Meier survival curves were analyzed using the log-rank test. Statistically significant correlations between HBXIP and Prdx1 expression in clinical tissues were determined using the chi-square test with GraphPad Prism software (GraphPad Software, CA). Each experiment was repeated at least three times.

### Reporting summary

Further information on research design is available in the Nature Research Reporting Summary linked to this article.

## Supplementary information

Supplementary Information

Reporting Summary

## Data Availability

The datasets generated and analyzed during the current study are available from the corresponding author, Dr. Xiaolei Zhou (email address: foxlei@live.cn), upon reasonable request, as described in the following figshare metadata record: 10.6084/m9.figshare.17088764. The full sequences of the newly constructed plasmids for this study are deposited in Addgene under the ID numbers listed in Supplementary Table 1.

## Code availability

No custom code was used to generate the data contained in this manuscript.
