# Supplementary material for: HBXIP induces anoikis resistance by forming a reciprocal feedback loop with Nrf2 to maintain redox homeostasis and stabilize Prdx1 in breast cancer
Source: NPJ Breast Cancer. 2022 Jan 13;8:7. doi: 10.1038/s41523-021-00374-x (PMC8758767; doi:10.1038/s41523-021-00374-x)
Supplement: Supplementary file 1 — Supplementary Information [file 41523_2021_374_MOESM1_ESM.pdf]

Supplementary Information

Supplementary Table 1. Key resources

REAGENT or RESOURCE	SOURCE		IDENTIFIER
**Antibodies**			
ANTI-β-actin (clone AC-74) Mouse mAb	WB: 1:2000	Sigma-Aldrich (St. Louis, MO, USA)	Cat# A2228, RRID: AB_476697
ANTI-FLAG® M2 Mouse mAb	WB: 1:2000	Sigma-Aldrich (St. Louis, MO, USA)	Cat# F1804, RRID: AB_262044
Anti-HBXIP (H-5) Mouse mAb	WB: 1:800<br>IHC: 1:100	Santa Cruz Biotechnology	Cat# sc-373980, RRID: AB_10918789
Anti-NRF2 (D1Z9C) XP® Rabbit mAb	WB: 1:1000<br>ChIP: 1:200	Cell Signaling Technology （Danvers, MA, USA）	Cat# 12721, RRID: AB_2715528
Rabbit (DA1E) mAb IgG XP® Isotype Control	ChIP: 1:200	Cell Signaling Technology （Danvers, MA, USA）	Cat# 3900, RRID: AB_1550038
Anti-Caspase-3 (3G2) Mouse mAb	WB: 1:1000	Cell Signaling Technology （Danvers, MA, USA）	Cat# 9668, RRID: AB_2069870
Anti-Caspase-7 (C7) Mouse mAb	WB: 1:1000	Cell Signaling Technology （Danvers, MA, USA）	Cat#9494, RRID: AB_2068141
Anti-PARP (46D11) Rabbit mAb	WB: 1:1000	Cell Signaling Technology （Danvers, MA, USA）	Cat# 9532, RRID: AB_659884
Anti-GST3/GSTpi Goat pAb	WB: 1:2500<br>IP: 1:200	Abcam (Cambridge, MA)	Cat# ab53943, RRID: AB_873819
Goat IgG Isotype Control	IP: 1:200	Invitrogen, Thermo Fisher Scientific, USA	Cat# 02-6202, RRID: AB_2532946
Anti-JNK1 Rabbit mAb	WB: 1:2000	Abcam (Cambridge, MA)	Cat# ab110724, RRID: AB_10866293
Anti-phospho-JNK1 Rabbit	WB: 1:1000	Abcam	Cat# ab46821, RRID: AB_881487

mAb		(Cambridge, MA)	
Anti-Peroxiredoxin 1 Mouse mAb	WB: ~0.1 μg/ml	R&D Systems (Minneapolis, MN, USA)	Cat# MAB3488, RRID: AB_2170319
Anti-E6AP Rabbit pAb	WB: 1:1000	Abcam (Cambridge, MA)	Cat# ab3519, RRID: AB_303868
Anti-Human Ubiquitin Rabbit pAb	WB: 1:1000	R&D Systems (Minneapolis, MN, USA)	Cat# A-100, RRID: AB_10971295
**Chemicals and reagents**			
N-Acetyl-L-cysteine, NAC	Sigma-Aldrich (St. Louis, MO, USA)		Cat# A7250
Human insulin	Sigma-Aldrich (St. Louis, MO, USA)		Cat# I9278
λ phosphatase	New England Biolabs (Ipswich, MA, USA)		Cat# P0753
MEM Non-Essential Amino Acids Solution	Invitrogen, Thermo Fisher Scientific, USA		Cat# 11140050
tert-Butylhydroquinone, tBHQ	Sigma-Aldrich (St. Louis, MO, USA)		Cat# 112941
ML385	Sigma-Aldrich (St. Louis, MO, USA)		Cat# SML1833
Polybrene	Sigma-Aldrich (St. Louis, MO, USA)		Cat# 107689
Poly-HEMA	Sigma-Aldrich (St. Louis, MO, USA)		Cat# P3932
Matrigel basement membrane-like matrix	Corning (Medfield, MA, USA)		Cat# 354234
TRIzol™ Reagent	Invitrogen, Thermo Fisher Scientific, USA		Cat# 15596018
Lipofectamine 2000	Invitrogen, Thermo Fisher Scientific, USA		Cat# 11668019
Lipofectamine™ RNAiMAX Transfection Reagent	Invitrogen, Thermo Fisher Scientific, USA		Cat# 13778075
Hydrogen peroxide	Thermo Fisher Scientific, USA		Cat# BSPA5
SP600125	MedChemExpress（Princeton, NJ, USA）		Cat# HY-12041
DB07268	MedChemExpress（Princeton, NJ, USA）		Cat# HY-15737
Age I	New England Biolabs (Ipswich, MA, USA)		Cat# R0552

EcoR I	New England Biolabs (Ipswich, MA, USA)	Cat# R0101
BamH I	New England Biolabs (Ipswich, MA, USA)	Cat# R0136
Xho I	New England Biolabs (Ipswich, MA, USA)	Cat# R0146
Hind III	New England Biolabs (Ipswich, MA, USA)	Cat# R0104
Bouin's fixative solution	Solarbio （Beijing, China）	Cat# G2331
DMEM (high glucose, with L-glutamine, without sodium pyruvate)	Hyclone (Logan, UT, USA)	Cat# SH30022
DMEM (high glucose, with L-glutamine and sodium pyruvate)	Hyclone (Logan, UT, USA)	Cat# SH30243
L-15 (with L-glutamine)	Hyclone (Logan, UT, USA)	Cat# SH30525
**Critical commercial assays**		
Annexin V-FITC-PI apoptosis detection kit	Vazyme Biotech (Nanjing, China)	Cat# KGA108
PrimeScript™ 1st Strand cDNA Synthesis Kit	TaKaRa Bio (Dalian, China)	Cat# 6110
GeneArt™ Site-directed Mutagenesis System	Invitrogen, Thermo Fisher Scientific, USA	Cat# A13282
Dual-Luciferase® Reporter Assay System	Promega (Madison, WI, USA)	Cat# E1910
Reactive oxygen species Assay Kit	Nanjing Jiancheng Bioengineering Institute （Nanjing, China）	Cat# E004-1
Coenzyme II （NADP /NADPH) content test kit	Nanjing Jiancheng Bioengineering Institute （Nanjing, China）	Cat# A115-1
Reduced glutathione (GSH) assay kit	Nanjing Jiancheng Bioengineering Institute （Nanjing, China）	Cat# A006-2
ATP assay kit	Nanjing Jiancheng Bioengineering Institute （Nanjing, China）	Cat# A095-2
Mitochondrial membrane potential detection kit	Nanjing Jiancheng Bioengineering Institute （Nanjing, China）	Cat# G009-1
Lenti-X™ HTX Packaging System	CLONTECH Laboratories, Inc., （Palo Alto, CA)	Cat# PT5135-2
NE-PER™ Nuclear and	Thermo Fisher Scientific, USA	Cat# 78833

Cytoplasmic Extraction Reagents		
Hematoxylin and Eosin Staining Kit	Beyotime Biothenology (Shanghai, China)	Cat# C0105
Protein G Immunoprecipitation Kit	Sigma-Aldrich (St. Louis, MO, USA)	Cat# IP50
TF Activation Profiling Plate Array II with Nuclear Extraction Kit	Signosis, Inc. （Sunnyvale, CA）	Cat# FA-1002-NE
QuikChIP ChIP Kit	Novus Biologicals (LLC., Littleton, CO, USA)	Cat# NBP2-29902
ProteinExt® Mammalian Total Protein Extraction Kit	Transgen (Beijing, China)	Cat# DE101-01
ProteinExt® Mammalian Nuclear and Cytoplasmic Protein Extraction Kit	Transgen (Beijing, China)	Cat# DE201-01
Hematoxylin and Eosin Stain Kit	Solarbio （Beijing, China）	Cat# G1121-4
Mouse Cell Depletion Kit	Miltenyi Biotec Inc. (Auburn, CA, USA)	Cat# 130-104-694
**Recombinant DNA**		
psPAX2	Laboratory of Xiaolei Zhou	N/A
pMD2.G	Laboratory of Xiaolei Zhou	N/A
pLKO.1-puro	Laboratory of Xiaolei Zhou	N/A
pLKO.1-neo	Laboratory of Xiaolei Zhou	N/A
pLVSIN-CMV pur	TaKaRa Bio (Shiga, Japan)	Cat# 6183
pLVSIN-CMV Hyg	TaKaRa Bio (Shiga, Japan)	Cat# 6182
pLVSIN-Control	Laboratory of Xiaolei Zhou	N/A
pLVSIN-HBXIP (neo)	Laboratory of Xiaolei Zhou	N/A
pLVSIN-Nrf2 (puro)	This paper	ID: 179281
pLVSIN-Prdx1 (hyg)	This paper	ID: 179295
pLKO-shControl	Laboratory of Xiaolei Zhou	N/A
pLKO-shHBXIP (puro)	Laboratory of Xiaolei Zhou	N/A
pLKO-shNrf2 (neo)	This paper	ID: 179300
pLKO-shPrdx1 (hyg)	This paper	ID: 179301
pRL-TK	Laboratory of Xiaolei Zhou	N/A
pCMV-SATB1	This paper	ID: 179302
pCMV-Nrf2	Laboratory of Xiaolei Zhou	N/A
pCMV-Sox2	This paper	ID: 179303
pCMV-HBXIP	Laboratory of Xiaolei Zhou	N/A
pGL3-HBXIP-FL	This paper	ID: 179304
pGL3-HBXIP-1688	This paper	ID: 179305
pGL3-HBXIP-1704	This paper	ID: 179306
pGL3-HBXIP-1025	This paper	ID: 179307

pGL3-HBXIP-808	This paper	ID: 179308
pGL3-NQO1-ARE	This paper	N/AID: 179313
pGL3-HBXIP-MT3	This paper	ID: 179309
pGL3-HBXIP-MT2	This paper	ID: 179310
pGL3-HBXIP-MT1	This paper	ID: 179311
pGL3-HBXIP-MT1/2	This paper	ID: 179312
**Recombinant lentiviruses**		
LV-Control	Laboratory of Xiaolei Zhou	N/A
LV-HBXIP (neo)	Laboratory of Xiaolei Zhou	N/A
LV-HBXIP (puro)	This paper	N/A
LV-Nrf2 (puro)	This paper	N/A
LV-Prdx1 (hyg)	This paper	N/A
LK-shControl	Laboratory of Xiaolei Zhou	N/A
LK-shHBXIP (puro)	Laboratory of Xiaolei Zhou	N/A
LK-shNrf2 (neo)	This paper	N/A
LK-shNrf2 (puro)	This paper	N/A
LK-Prdx1 (hyg)	This paper	N/A

Supplementary Table 2. Primers used in this study

Name	Primer	Sequence
**For cDNA cloning**		
Nrf2	Forward	5'-CTCGAGATGATGGACTTGGAGCTGCC-3'
	Reverse	5'-GGATCCCTAAATCTAGTTTTTCTTAACATCTGGCTTC-3'
SATB1	Forward	5'-GGATCCATGGATCATTTGAACGAGGC-3'
	Reverse	5'-AAGCTTATCTCAGTCTTTCAAATCAGTATTAATGTC-3'
Sox2	Forward	5'-GGATCCATGTACAACATGATGGAGACGGAG-3'
	Reverse	5'-AAGCTTGGCCCTCACATGTGTGAGAG-3'
Prdx1	Forward	5'-GGATCCATGTCTTCAGGAAATGCTAAAATTGGGC-3'
	Reverse	5'-AAGCTTTCACTTCTGCTTGGAGAAATATTCTTTGC-3'
**For promoter cloning**		
HBXIP-FL	Forward	5'-CGGGTACCCACCAGGCAGTACTTATGTTTTCTAATG-3'
	Revers	5'-CGACGCGTGAGGCGCGCACTACTCACGT-3'

	Reverse	
HBXIP-1688	Forward	5’-CGGGTACCCAAGGAAGCTGCAAGCTTCA-3’
	Reverse	5’-CGACGCGTGAGGCGCGCACTACTCACGT-3’
HBXIP-1074	Forward	5’-CGGGTACCGAACCTAGAAGGCTGAGGCT-3’
	Reverse	5’-CGACGCGTGAGGCGCGCACTACTCACGT-3’
HBXIP-1025	Forward	5’-CGGGTACCCCAACCTTAATCCACAATTAAGATACA-3’
	Reverse	5’-CGACGCGTGAGGCGCGCACTACTCACGT-3’
HBXIP-808	Forward	5’-CGGGTACCCACATTTTGGGAGGCTGGA-3’
	Reverse	5’-CGACGCGTGAGGCGCGCACTACTCACGT-3’
NQO1-ARE	Forward	5’-GGGGCTAGCTCCGGGTTCAAGCGATTC-3’
	Reverse	5’-CCCCCGGGGCTCTGGTGCAGTCCGG-3’
**For promoter mutation**		
HBXIP-MT1	Forward	5‘-CAAAAAAAACCATGCTAGGCGTGTGTCAGACTAATGTAATCACATTTTGGGAGG-3’
	Reverse	5‘-CCTCCCAAAATGTGATTACATTAGTCTGACACACGCCTAGCATGGTTTTTTTTG-3’
HBXIP-MT2	Forward	5‘-GAAGGCTGAGGCTGGACTAAGCCCGTCGCTGTAAACTGCACTCCAACCTTAATCCAC-3’
	Reverse	5‘-GTGGATTAAGGTTGGAGTGCAGTTTACAGCGACGGGCTTAGTCCAGCCTCAGCCTTC-3’
HBXIP-MT3	Forward	5‘-CAGGAGGCAGAGGTTGCAGTGAGAGTCTCATTATCCACTGCACTCCAGCCTGGGCG-3’
	Reverse	5‘-CGCCCAGGCTGGAGTGCAGTGGATAATGAGACTCTCACTGCAACCTCTGCCTCCTG-3’
**For ChIP-qRT-PCR**		
GAPDH	Forward	5’-TCCTGCACCACCAACTGCTTAG-3’
	Reverse	5’-GATGACCTTGCCCACAGCCTTG-3’
NQO1-ARE	Forward	5’-CAGTGGCATGCACCCAGGGAA-3’
	Reverse	5’-GCATGCCCCTTTTAGCCTTGGCA-3’

HBXIP-ARE 1	Forward	5'-GTACCTCCAGTATGTATGTCAATTTTGC-3'
	Reverse	5'-TGGTCTTAAACTCCTTGCTTCAACAG-3'
HBXIP-ARE 2	Forward	5'-AAGATCGCTTGAACCTAGAAGGC-3'
	Reverse	5'-GTTTTCACTTACATCCTTTGTAGTAGTT-3'
HBXIP-ARE 3	Forward	5'-GTAATCCCAGCTACTCGGGA-3'
	Reverse	5'-GAGACAGAGTCTTGCTCTATCGC-3'
**For qRT-PCR**		
HBXIP	Forward	5'-CTTGGAGCAGCACTTGGAAGA-3'
	Reverse	5'-ATGCCATCGTGTTTCTGGATC-3'
GAPDH	Forward	5'-TCCTGCACCACCAACTGCTTAG-3'
	Reverse	5'-GATGACCTTGCCCACAGCCTTG-3'
**For RT-PCR**		
Prdx1 (for RT-PCR)	Forward	5'-GTCCCACGGAGATCATTGCTTTC-3'
	Reverse	5'-CCCCTGAAAGAGATACCTTCATC-3'
β-actin (for RT-PCR)	Forward	5'-ACTTGCGCTCAGGAGGAGCAATG-3'
	Reverse	5'-GAGATGGCCACTGCCGCATCCTCTT-3'

Supplementary Table 3. Cell lines used in this study

Cell	SOURCE	RRID	Medium	Serum	Additive	CO_2
Human: MCF-7	ATCC, Laboratory of Xiaolei Zhou	Cat# HTB-22TM, Lot# 60012672, RRID: CVCL_0031	DMEM	10% FBS	1% s/p	5%
Human: MDA-MB-436	ATCC, Laboratory of	Cat# HTB-130,	L-15	10% FBS	10 μg/ml insulin, 1% s/p	-

	Xiaolei Zhou	Lot# 61331460 , RRID: CVCL_0623				
Human：HEK293	ATCC , Laboratory of Xiaolei Zhou	Cat# CRL-1573™, Lot# 7681666 , RRID: CVCL_0045	DMEM	10% FBS	1% s/p	5%
Human： 293T	ATCC , Laboratory of Xiaolei Zhou	Cat# CRL-3216, RRID: CVCL_0063	DMEM	10% FBS	1% s/p	5%
Human： MCF-HBXIP	Laboratory of Xiaolei Zhou	N/A	DMEM	10% FBS	1% s/p, 200 μg/ml G418	5%
Human : MDA-MB-436-HBXIP KD	Laboratory of Xiaolei Zhou	N/A	L-15	10% FBS	10 μg/ml insulin, 1% s/p, 2 μg/ml puromycin	-
Human: MCF-Nrf2 KD	This paper	N/A	DMEM	10% FBS	1% s/p, 200 μg/ml G418	5%
Human：MCF-Nrf2	This paper	N/A	DMEM	10% FBS	1% s/p, 2 μg/ml puromycin	5%
Human : MDA-MB-436-Nrf2 KD	This paper	N/A	L-15	10% FBS	10 μg/ml insulin, 1% s/p, 2 μg/ml puromycin	-
Human： MCF-HBXIP/Nrf2	This paper	N/A	DMEM	10% FBS	1% s/p, 2 μg/ml puromycin, 200 μg/ml G418	5%
Human: MDA-MB-436-HBXIP/Nrf2 KD	This paper	N/A	L-15	10% FBS	10 μg/ml insulin, 1% s/p, 2 μg/ml puromycin, 200 μg/ml G418	-
Human : MCF-HBXIP OE/Nrf2 KD	This paper	N/A	DMEM	10% FBS	1% s/p, 2 μg/ml puromycin, 200 μg/ml	5%

					G418	
Human : MCF-HBXIP KD/Nrf2 OE	This paper	N/A	DMEM	10% FBS	1% s/p, 2 µg/ml puromycin, 200 µg/ml G418	5%
Human: MDA-MB-436-HBXIP OE/Nrf2 KD	This paper	N/A	L-15	10% FBS	10 µg/ml insulin, 1% s/p, 2 µg/ml puromycin, 200 µg/ml G418	-
Human: MDA-MB-436-HBXIP KD/Nrf2 OE	This paper	N/A	L-15	10% FBS	10 µg/ml insulin, 1% s/p, 2 µg/ml puromycin, 200 µg/ml G418	
Mouse: MEF wt	Laboratory of Xiaolei Zhou	N/A	DMEM	10% FBS	1% non-essential amino acids, 5 mM glutamine, 1% s/p	5%
Mouse: *HBXIP*$^{-/-}$ MEF	Laboratory of Xiaolei Zhou	N/A	DMEM	10% FBS	1% non-essential amino acids, 1% s/p, 5 mM glutamine, 2 µg/ml puromycin	5%
Mouse: Nrf2$^{-/-}$ MEF	Tohoku University, Japan	N/A	DMEM	10% FBS	1% non-essential amino acids, 5 mM glutamine, 1% s/p	5%

Supplementary Table 4. siRNA sequences

**Name**	**Sequence**
siHBXIP-1	5'-CGGAAGCGCAGUGAUGUUU-3'
siHBXIP-2	5'-CAGAGGUUAGCUUAGCUGC-3'

siNrf2-1	5'- AGUUGAGCUUCAUUGAACUGC-3'
siNrf2-2	5'- GGUUGAGACUACCAUGGUU-3'

Supplementary Table 5. Probe sequences used for EMSAs

EMSA probe (30 bp)	Sequence
ARE1	5'- CTAGGCGTGGTGACTCAGCCTGTAATCACA-3'
ARE1mut	5'- CTAGGCGTG**AGAGA**TCAGCCTGTAATCACA-3' (Bold, mutant sites)
ARE2	5'- GACTAAGCCATGATAGTGCCACTGCACTCC-3'
ARE3	5'- GCAGTGAGCTGAGACGGCGCCACTGCACTC-3'

Supplementary Table 6. Clinical characteristics of the breast tissues, including breast carcinoma tissue, carcinoma metastasized to the lymph nodes, and normal tissue samples.

Age	Sex	Organ/Anatomical Site	Pathological diagnosis	TNM	Grade	Stage	Type
38	F	Breast	Adjacent normal breast tissue（adenosis)	-	-	-	NAT
48	F	Breast	Adjacent normal breast tissue（adenosis)	-	-	-	NAT
41	F	Breast	Adjacent normal breast tissue（adenosis)	-	-	-	NAT
49	F	Breast	Adjacent normal breast tissue（adenosis)	-	-	-	NAT
37	F	Breast	Adjacent normal breast tissue（adenosis)	-	-	-	NAT
46	F	Breast	Adjacent normal breast tissue	-	-	-	NAT
41	F	Breast	Adjacent normal breast tissue（adenosis)	-	-	-	NAT
41	F	Breast	Adjacent normal breast tissue	-	-	-	NAT
53	F	Breast	Adjacent normal breast tissue（adenosis)	-	-	-	NAT
40	F	Breast	Adjacent normal breast tissue（adenosis)	-	-	-	NAT
35	F	Breast	Adjacent normal breast tissue	-	-	-	NAT
47	F	Breast	Adjacent normal breast tissue（adenosis)	-	-	-	NAT

42	F	Breast	Adjacent normal breast tissue（adenosis）	-	-	-	NAT
44	F	Breast	Adjacent normal breast tissue（adenosis）	-	-	-	NAT
46	F	Breast	Adjacent normal breast tissue（adenosis）	-	-	-	NAT
29	F	Breast	Adjacent normal breast tissue	-	-	-	NAT
50	F	Breast	Adjacent normal breast tissue（adenosis）	-	-	-	NAT
43	F	Breast	Adjacent normal breast tissue（adenosis）	-	-	-	NAT
60	F	Breast	Adjacent normal breast tissue	-	-	-	NAT
46	F	Breast	Adjacent normal breast tissue（adenosis）	-	-	-	NAT
55	F	Breast	Invasive ductal carcinoma	T2N0M0	2	IIA	Malignant
32	F	Breast	Invasive ductal carcinoma	T2N0M0	2	IIB	Malignant
59	F	Breast	Invasive ductal carcinoma	T2N0M0	2	I	Malignant
87	F	Breast	Invasive ductal carcinoma	T2N1M0	2	I	Malignant
67	F	Breast	Invasive ductal carcinoma	T2N0M0	3	IIIB	Malignant
45	F	Breast	Invasive ductal carcinoma	T2N2M0	2	IIB	Malignant
45	F	Breast	Invasive ductal carcinoma	T2N0M0	2	IIA	Malignant
58	F	Breast	Invasive ductal carcinoma	T3N0M0	2	IIA	Malignant
46	F	Breast	Invasive ductal carcinoma	T2N1M0	2	IIA	Malignant
54	F	Breast	Invasive ductal carcinoma	T1N0M0	2	IIB	Malignant
50	F	Breast	Invasive ductal carcinoma	T2N0M0	2	IIA	Malignant
69	F	Breast	Invasive ductal carcinoma	T2N0M0	3	IIA	Malignant
44	F	Breast	Invasive ductal carcinoma	T2N0M0	2	IIA	Malignant
52	F	Breast	Invasive ductal carcinoma	T2N0M0	2	IIA	Malignant
38	F	Breast	Invasive ductal carcinoma	T2N0M0	2	IIA	Malignant
41	F	Breast	Invasive ductal carcinoma	T2N0M0	2	IIA	Malignant
47	F	Breast	Invasive ductal carcinoma	T4N0M0	3	I	Malignant
64	F	Breast	Invasive ductal carcinoma	T2N0M0	2	IIB	Malignant
56	F	Breast	Invasive ductal carcinoma	T1N0M0	2	IIA	Malignant
95	F	Breast	Invasive ductal carcinoma	T2N0M0	2	IIA	Malignant
52	F	Breast	Invasive ductal carcinoma	T2N0M0	2	IIB	Malignant
48	F	Breast	Invasive ductal carcinoma	T2N0M0	2	IIA	Malignant
49	F	Breast	Invasive ductal carcinoma	T2N1M0	2	IIA	Malignant
52	F	Breast	Invasive ductal carcinoma	T2N0M0	2	IIB	Malignant
38	F	Breast	Invasive ductal carcinoma	T2N1M0	2	IIA	Malignant
44	F	Breast	Invasive ductal carcinoma	T2N0M0	2	IIA	Malignant
43	F	Breast	Invasive ductal carcinoma	T2N0M0	2	IIIB	Malignant

46	F	Breast	Invasive ductal carcinoma	T2N0M0	3	IIA	Malignant
74	F	Breast	Invasive ductal carcinoma	T1N0M0	2	IIIB	Malignant
50	F	Breast	Invasive ductal carcinoma	T4N0M0	2	IIIB	Malignant
71	F	Breast	Invasive ductal carcinoma	T1N0M0	1	IIA	Malignant
42	F	Breast	Invasive ductal carcinoma	T2N1M0	2	IIIA	Malignant
48	F	Breast	Invasive ductal carcinoma	T1N0M0	2	IIA	Malignant
49	F	Breast	Invasive ductal carcinoma	T2N0M0	2	IIA	Malignant
45	F	Breast	Invasive ductal carcinoma	T4N2M0	2	IIA	Malignant
50	F	Breast	Invasive ductal carcinoma	T2N0M0	2	I	Malignant
44	F	Breast	Invasive ductal carcinoma	T2N0M0	2	IIA	Malignant
36	F	Breast	Invasive ductal carcinoma	T3N0M0	1	IIA	Malignant
42	F	Breast	Invasive ductal carcinoma	T2N1M0	2	IIA	Malignant
48	F	Breast	Invasive ductal carcinoma	T3N0M0	2	I	Malignant
39	F	Breast	Invasive ductal carcinoma	T2N0M0	2	IIB	Malignant
42	F	Breast	Invasive ductal carcinoma	T2N0M0	3	IIA	Malignant
50	F	Breast	Invasive ductal carcinoma	T2N0M0	2	IIA	Malignant
36	F	Breast	Invasive ductal carcinoma	T2N1M0	2	IIA	Malignant
50	F	Breast	Invasive ductal carcinoma	T2N0M0	3	IIA	Malignant
46	F	Breast	Invasive ductal carcinoma	T2N0M0	2	IIB	Malignant
38	F	Breast	Invasive ductal carcinoma	T2N0M0	2	IIB	Malignant
45	F	Breast	Invasive ductal carcinoma	T4N0M0	3	IIA	Malignant
41	F	Breast	Invasive ductal carcinoma	T2N0M0	2	IIA	Malignant
43	F	Breast	Invasive ductal carcinoma	T3N1M0	2	IIA	Malignant
68	F	Breast	Invasive ductal carcinoma	T2N0M0	3	IIA	Malignant
52	F	Breast	Invasive ductal carcinoma	T3N0M0	2	IIA	Malignant
45	F	Breast	Invasive ductal carcinoma	T2N0M0	2	IIIA	Malignant
47	F	Breast	Invasive ductal carcinoma	T1N1M0	2	IIB	Malignant
46	F	Breast	Invasive ductal carcinoma	T2N0M0	2	IIA	Malignant
61	F	Breast	Invasive ductal carcinoma	T2N0M0	2	IIA	Malignant
37	F	Breast	Invasive ductal carcinoma	T2N0M0	2	IIA	Malignant
66	F	Breast	Invasive ductal carcinoma	T2N0M0	1	IIIB	Malignant
46	F	Breast	Invasive ductal carcinoma	T4N0M0	2	IIB	Malignant
39	F	Breast	Invasive ductal carcinoma	T4N1M0	1	IIIA	Malignant
51	F	Breast	Invasive ductal carcinoma	T2N0M0	2	IIIB	Malignant
47	F	Breast	Invasive ductal carcinoma	T2N0M0	2	IIA	Malignant
42	F	Breast	Invasive ductal carcinoma	T2N0M0	2	IIA	Malignant
48	F	Breast	Invasive ductal carcinoma	T4N0M0	2	IIIB	Malignant
38	F	Breast	Invasive ductal carcinoma	T2N0M0	2	IIA	Malignant
62	F	Breast	Invasive ductal carcinoma	T2N0M0	2	IIB	Malignant
35	F	Breast	Invasive ductal carcinoma	T2N0M0	2	IIA	Malignant
59	F	Breast	Invasive ductal carcinoma	T2N1M0	3	IIA	Malignant
38	F	Breast	Invasive ductal carcinoma	T3N1M0	2	IIA	Malignant
55	F	Breast	Invasive ductal carcinoma	T2N1M0	2	IIB	Malignant

43	F	Lymph node	Metastatic invasive ductal carcinoma	-	2	-	Metastasis
55	F	Lymph node	Metastatic invasive lobular carcinoma	-	2	-	Metastasis
48	F	Lymph node	Metastatic invasive ductal carcinoma	-	2	-	Metastasis
51	F	Lymph node	Metastatic invasive ductal carcinoma	-	3	-	Metastasis
69	F	Lymph node	Metastatic invasive ductal carcinoma	-	2	-	Metastasis
38	F	Lymph node	Metastatic invasive ductal carcinoma	-	3	-	Metastasis
37	F	Lymph node	Metastatic invasive ductal carcinoma	-	3	-	Metastasis
32	F	Lymph node	Metastatic invasive ductal carcinoma	-	3	-	Metastasis
51	F	Lymph node	Metastatic invasive ductal carcinoma	-	2	-	Metastasis
51	F	Lymph node	Metastatic invasive lobular carcinoma	-	2	-	Metastasis
56	F	Lymph node	Metastatic invasive ductal carcinoma	-	2	-	Metastasis
71	F	Lymph node	Metastatic invasive ductal carcinoma	-	-	-	Metastasis
47	F	Lymph node	Metastatic invasive ductal carcinoma	-	2	-	Metastasis
43	F	Lymph node	Metastatic invasive ductal carcinoma	-	2	-	Metastasis
55	F	Lymph node	Metastatic invasive ductal carcinoma	-	2	-	Metastasis
51	F	Lymph node	Metastatic invasive ductal carcinoma	-	1	-	Metastasis
58	F	Lymph node	Metastatic invasive ductal carcinoma	-	2	-	Metastasis
49	F	Lymph node	Metastatic invasive ductal carcinoma	-	2	-	Metastasis
28	F	Lymph node	Metastatic invasive ductal carcinoma	-	2	-	Metastasis
51	F	Lymph node	Metastatic invasive ductal carcinoma	-	3	-	Metastasis
52	F	Lymph node	Metastatic invasive ductal carcinoma	-	2	-	Metastasis
35	F	Lymph node	Metastatic invasive ductal	-	2	-	Metastasis

			carcinoma				
52	F	Lymph node	Metastatic invasive ductal carcinoma	-	2	-	Metastasis
59	F	Lymph node	Metastatic invasive ductal carcinoma	-	-	-	Metastasis
51	F	Lymph node	Metastatic invasive ductal carcinoma	-	2	-	Metastasis
50	F	Lymph node	Metastatic invasive ductal carcinoma	-	2	-	Metastasis
42	F	Lymph node	Metastatic invasive ductal carcinoma	-	1	-	Metastasis
36	F	Lymph node	Metastatic invasive ductal carcinoma	-	2	-	Metastasis
44	F	Lymph node	Metastatic invasive ductal carcinoma	-	2	-	Metastasis
52	F	Lymph node	Metastatic invasive ductal carcinoma	-	1	-	Metastasis
50	F	Lymph node	Metastatic invasive ductal carcinoma	-	2	-	Metastasis
58	F	Lymph node	Metastatic invasive ductal carcinoma	-	1	-	Metastasis
57	F	Lymph node	Metastatic invasive lobular carcinoma	-	2	-	Metastasis
43	F	Lymph node	Metastatic invasive ductal carcinoma	-	2	-	Metastasis
68	F	Lymph node	Metastatic invasive ductal carcinoma	-	2	-	Metastasis
59	F	Lymph node	Metastatic invasive ductal carcinoma	-	2	-	Metastasis
70	F	Lymph node	Metastatic invasive ductal carcinoma	-	2	-	Metastasis
46	F	Lymph node	Metastatic invasive ductal carcinoma	-	2	-	Metastasis

Supplementary Table 7：Clinical characteristics of breast invasive carcinoma (TCGA, PanCancer Atlas) patients with complete data demographics

Cancer Type Detailed	Number	Percentage
Breast Invasive Ductal Carcinoma	741	74.50%
Breast Invasive Lobular Carcinoma	162	16.30%
Breast Invasive Carcinoma (NOS)	71	7.10%

Breast Invasive Mixed Mucinous Carcinoma	13	1.30%
Metaplastic Breast Cancer	6	0.60%
Invasive Breast Carcinoma	1	<0.1%
**Subtype**	**Number**	**Percentage**
BRCA_LumA	499	50.20%
BRCA_LumB	197	19.80%
BRCA_Basal	171	17.20%
NA	13	1.30%
BRCA_Her2	78	7.80%
BRCA_Normal	36	3.60%
**Diagnosis Age**	**Number**	**Percentage**
⩽30	12	1.21%
30-40	80	8.05%
40-50	207	20.82%
50-60	253	25.45%
60-70	257	25.86%
70-80	137	13.78%
80-90	48	4.83%
**Race Category**	**Number**	**Percentage**
White	687	69.10%
Black or African American	162	16.30%
NA	85	8.60%
Asian	59	5.90%
American Indian or Alaska Native	1	0.10%
**Neoplasm Disease Stage （American Joint Committee on Cancer Code）**		
STAGE IIB	234	23.50%
STAGE IIIA	139	14.00%
STAGE I	83	8.40%
STAGE IA	78	7.80%
STAGE IIIC	51	5.10%
STAGE IIIB	26	2.60%
STAGE IV	18	1.80%
STAGE X	13	1.30%
STAGE II	6	0.60%
STAGE IB	5	0.50%
NA	5	0.50%
STAGE III	2	0.20%
**Total**	**994**	

Supplementary Figures and Legends

Supplementary Figure S1

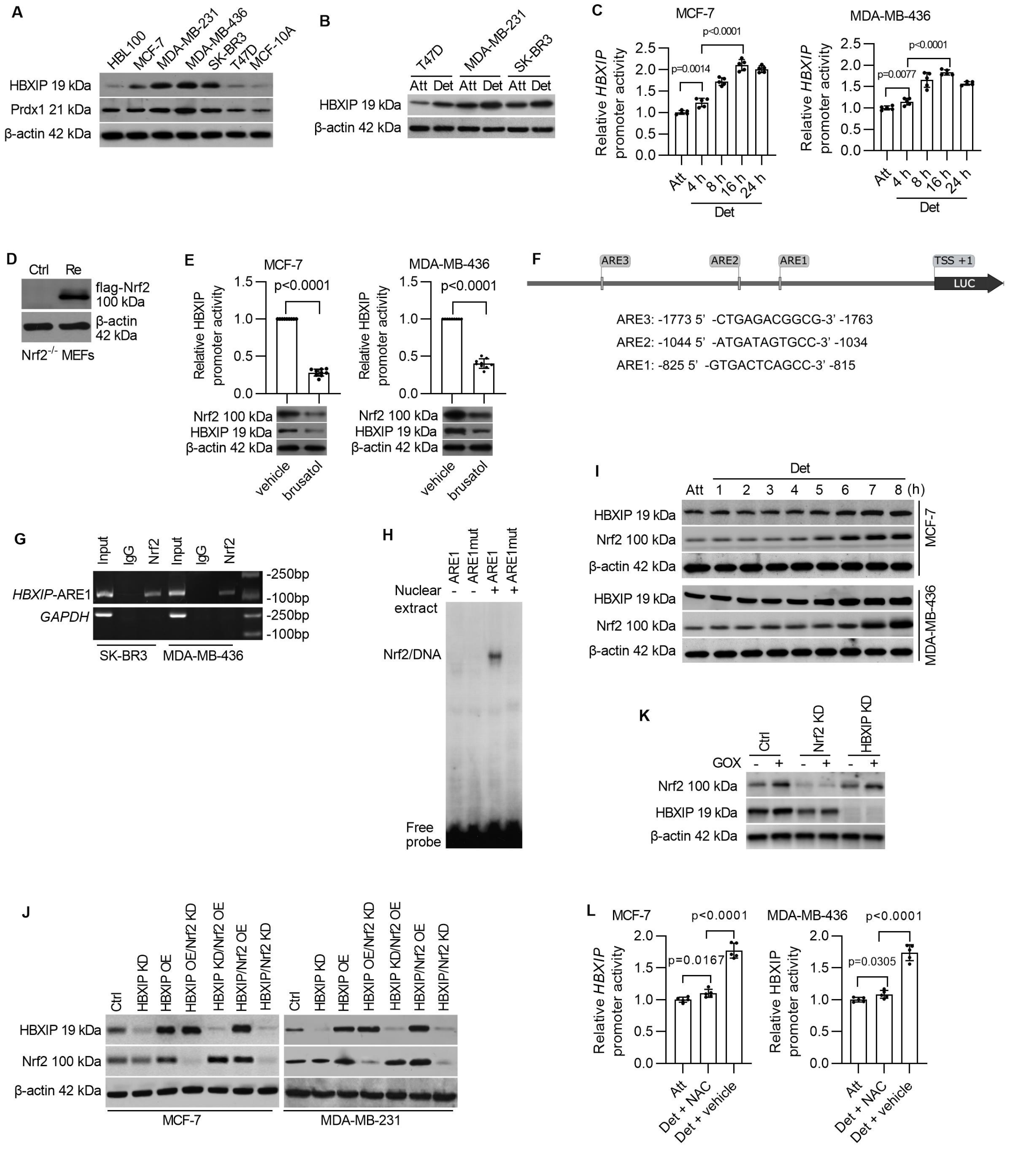

Figure S1. (A) HBXIP and Prdx1 protein levels in the indicated breast cancer cells and normal breast epithelial cells were measured using western blot analysis. (B) The expression level of HBXIP in T47D, MDA-MB-231 and SK-BR3 cells cultured in attachment and suspension was detected using western

blot analysis. (C) *HBXIP* promoter activity was investigated in MCF-7 and MDA-MB-436 cells, which were detached and cultured in suspension in poly-HEMA-coated plates for the indicated times. (D) The "rescued" expression of flag-Nrf2 in Nrf2 knockout MEFs was detected using western blotting. (E) After treatment of MCF-7 and MDA-MB-436 cells with 40 nM brusatol, a specific Nrf2 inhibitor, for 4 h, changes in HBXIP promoter activity were detected. Western blot shows the expression of HBXIP and Nrf2 in the indicated cells. F) Putative cis-acting AREs were analyzed using the Cistrome Analysis Pipeline (http://cistrome.org/). (G) SK-BR3 and MDA-MB-436 cells detached for 8 h were fixed with formaldehyde and cross-linked, and chromatin was sheared ultrasonically. Chromatin was immunoprecipitated with an anti-Nrf2 rabbit monoclonal antibody (mAb) or rabbit (DA1E) control IgG mAb. Nrf2 binding to the *HBXIP* promoter was analyzed via PCR with primers specific for the ARE1 region in the *HBXIP* promoter. GAPDH primers were used as a negative control. The ARE1 region in the *HBXIP* promoter was amplified from 5 µl of purified soluble chromatin before immunoprecipitation for use as input DNA. (H) *HBXIP* ARE1 and ARE1mut (Supplementary Table 5) were end-labeled with [γ-^{32}P]ATP and T4 kinase. Labeled DNA (100,000 cpm) was incubated with 10 µg of MCF-7 nuclear extract in binding buffer. The reaction mixtures were separated on a polyacrylamide gel and autoradiographed. DNA-protein complexes containing Nrf2 are indicated. (I) The expression of HBXIP and Nrf2 in suspended cultured MCF-7 and MDA-MB-436 cells was detected in an increasing time course. (J) HBXIP and Nrf2 expression in the indicated stable breast cancer cell lines. (K) Nrf2 and HBXIP expression in HBXIP- and Nrf2-deficient MCF-7 cells treated with glucose oxidase (GOX) for 2 h. (L) *HBXIP* promoter activity was investigated in MCF-7 and MDA-MB-436 cells in detached culture for 8 hours after treatment with 5 mM NAC. The error bars indicate the ±SD values assessed determined using Student's *t* test. All experiments were performed at least 3 times.

Supplementary Figure S2

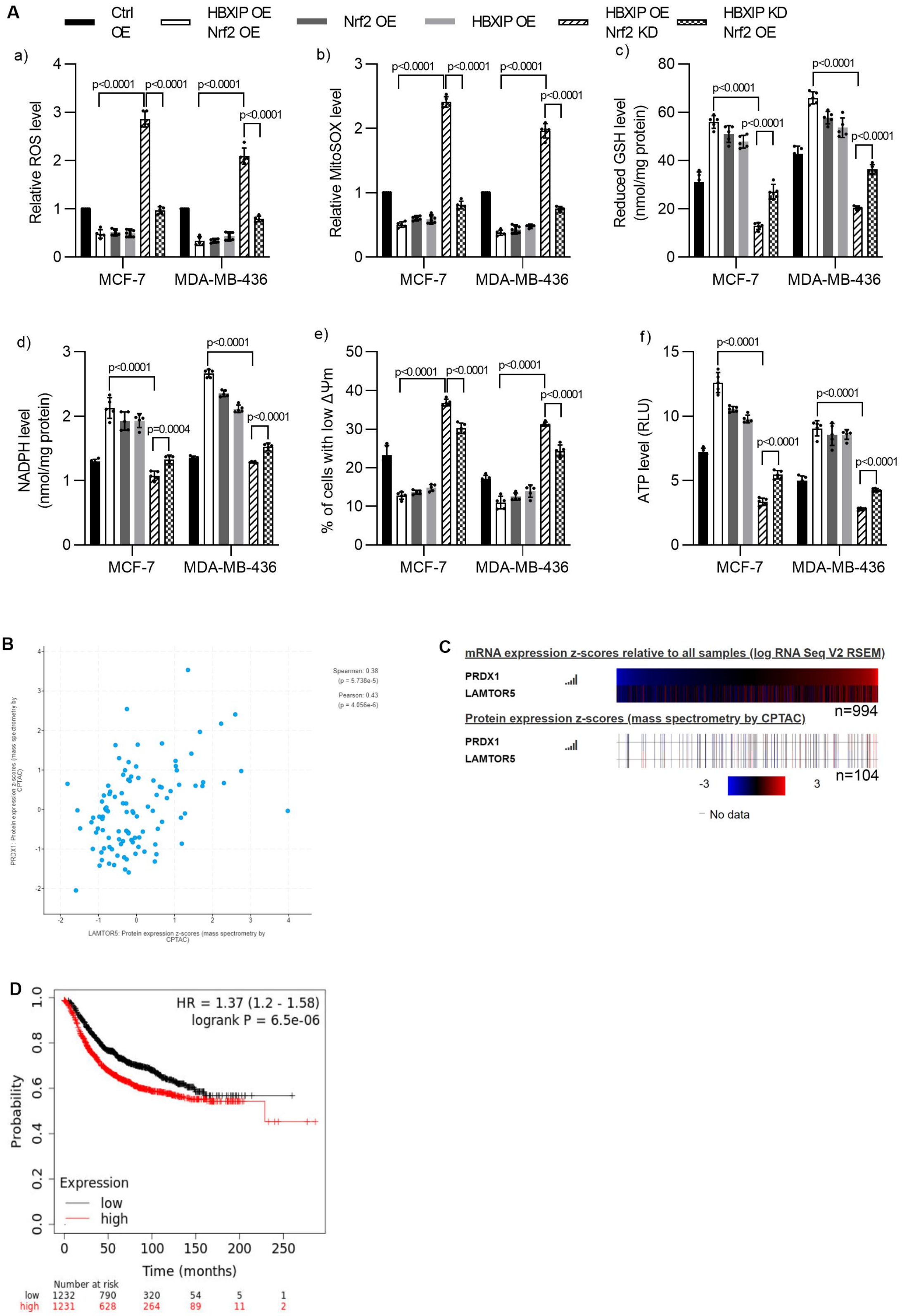

Figure S2. (A) The indicated stable MCF-7 and MDA-MB-436 cell lines cultured in suspension for 48 h

were a) stained with CM-H_2DCFDA and subjected to flow cytometry to determine the intracellular ROS level; b) stained with MitoSOX Red, and the ROS levels were determined; c) used to measure the reduced GSH levels; d) used to measure NADPH levels; e) used to assess the mitochondrial membrane potential using a JC1 assay; f) used to measure ATP with an ATP determination kit. (B) HBXIP and Prdx1 protein expression levels (z scores, mass spectrometry by CPTAC) were analyzed in 104 eligible clinical breast cancer samples (Pearson's correlation coefficient; r = 0.43, Spearman's correlation coefficient = 0.38). C Heatmaps showing the correlation between Prdx1 and HBXIP (*lamtor5*) mRNA expression (upper panel) and protein expression (lower panel). (D) Breast cancer patients (2463) filtered by high HBXIP expression on Kaplan-Meier Plotter platforms. A recurrence-free survival (RFS) curve of the HBXIP high/Prdx1 high group and HBXIP high/Prdx1 low group was generated using the Kaplan–Meier method and compared statistically using a log rank test. The error bars indicate the ± SD values as assessed by Student's *t* test. All the experiments were performed at least 3 times.

Supplementary Figure S3

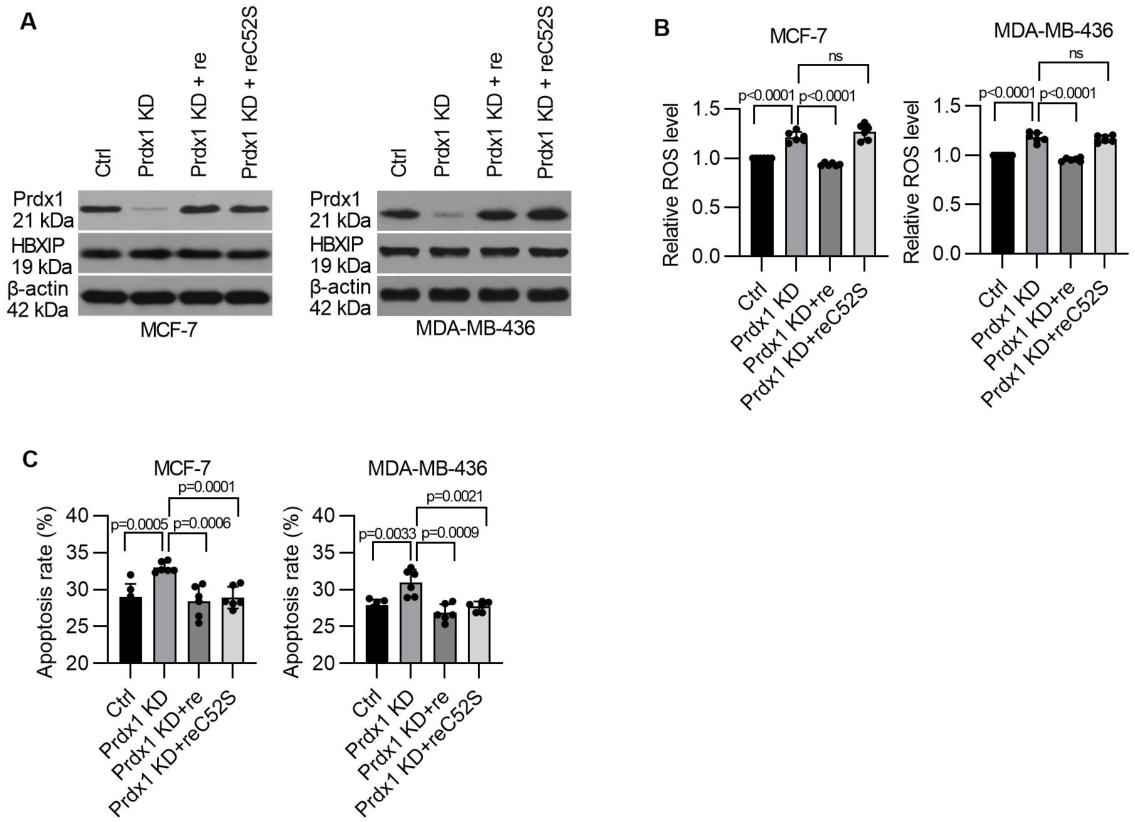

Figure S3. (A) The "rescued" expression of Prdx1 and Prdx1 C52S mutant in Prdx1-deficient MCF-7 and MDA-MB-436 cells was detected using western blotting. (B) The indicated cells in (A) cultured in suspension for 48 h were stained with CM-H_2DCFDA and subjected to flow cytometry to determine the intracellular ROS level. (C) The indicated cells in (A) cultured in suspension for 48 h were stained with Annexin V and subjected to flow cytometry to determine the apoptosis rate. The error bars indicate the ± SD values as assessed by Student's *t* test. All the experiments were performed at least 3 times.

Supplementary Figure S4

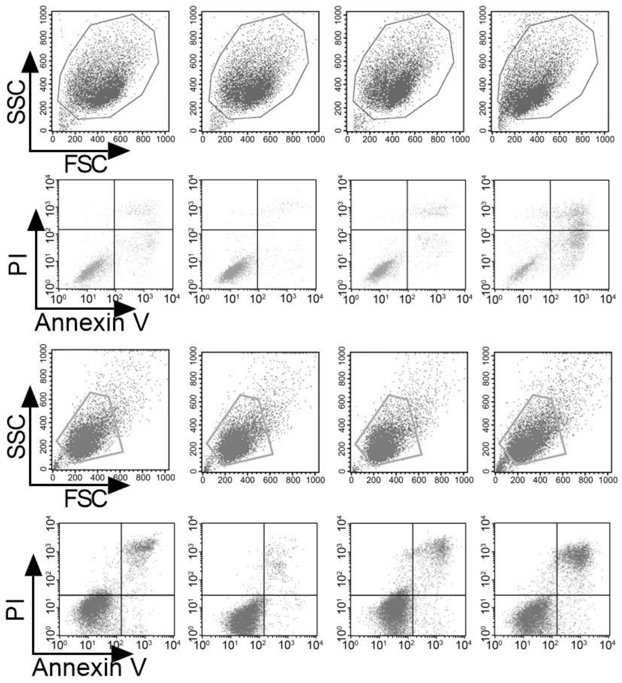

Figure S4: FSC/SSC plot and gating strategies of Figure1D

Supplementary Figure S5

Figure 1A left panel, HBXIP

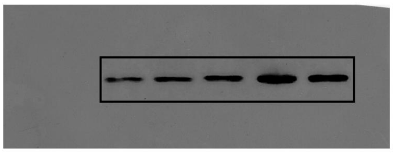

Figure 1A Right panel, HBXIP

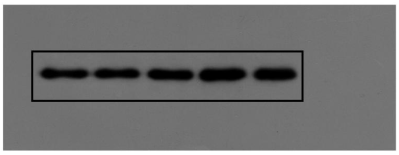

Figure 1F left panel, Caspase-7 and Cleaved Caspase-7

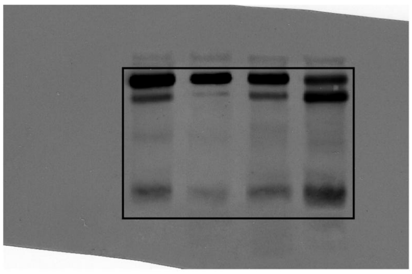

Figure 1F Right panel, Caspase-3 and Cleaved Caspase-3

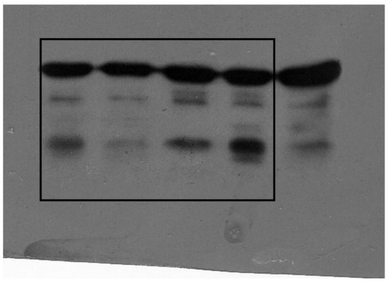

Figure 5C, Right panel, Prdx1

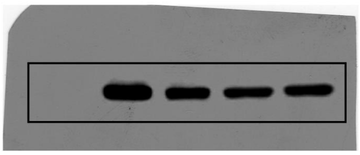

Figure 5D, Right panel, Prdx1

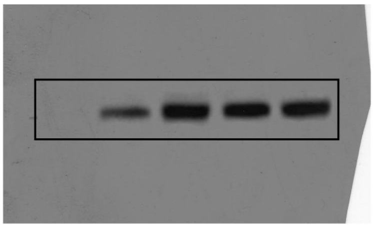

Figure 5C, Lift panel, JNK1

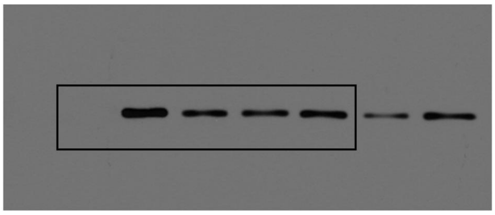

Figure 5D, Lift panel, JNK1

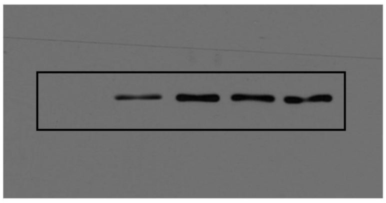

Figure S5: Uncropped western blot images related to the indicated important blots.
