# Supplementary material for: HBXIP induces anoikis resistance by forming a reciprocal feedback loop with Nrf2 to maintain redox homeostasis and stabilize Prdx1 in breast cancer
Source: NPJ Breast Cancer. 2022 Jan 13;8:7. doi: 10.1038/s41523-021-00374-x (PMC8758767; doi:10.1038/s41523-021-00374-x)
Supplement: Supplementary file 2 — Reporting Summary [file 41523_2021_374_MOESM2_ESM.pdf]

nature portfolio

Corresponding author(s): DBPR NPJBCANCER-01295R1 Xiaolei Zhou

Last updated by author(s): Nov 30, 2021

Reporting Summary

Nature Portfolio wishes to improve the reproducibility of the work that we publish. This form provides structure for consistency and transparency in reporting. For further information on Nature Portfolio policies, see our Editorial Policies and the Editorial Policy Checklist.

Statistics

For all statistical analyses, confirm that the following items are present in the figure legend, table legend, main text, or Methods section.

n/a	Confirmed	
☐	☒	The exact sample size (n) for each experimental group/condition, given as a discrete number and unit of measurement
☐	☒	A statement on whether measurements were taken from distinct samples or whether the same sample was measured repeatedly
☐	☒	The statistical test(s) used AND whether they are one- or two-sided<br>*Only common tests should be described solely by name; describe more complex techniques in the Methods section.*
☒	☐	A description of all covariates tested
☐	☒	A description of any assumptions or corrections, such as tests of normality and adjustment for multiple comparisons
☐	☒	A full description of the statistical parameters including central tendency (e.g. means) or other basic estimates (e.g. regression coefficient) AND variation (e.g. standard deviation) or associated estimates of uncertainty (e.g. confidence intervals)
☐	☒	For null hypothesis testing, the test statistic (e.g. F, t, r) with confidence intervals, effect sizes, degrees of freedom and P value noted<br>*Give P values as exact values whenever suitable.*
☒	☐	For Bayesian analysis, information on the choice of priors and Markov chain Monte Carlo settings
☒	☐	For hierarchical and complex designs, identification of the appropriate level for tests and full reporting of outcomes
☐	☒	Estimates of effect sizes (e.g. Cohen's d, Pearson's r), indicating how they were calculated

Our web collection on statistics for biologists contains articles on many of the points above.

Software and code

Policy information about availability of computer code

Data collection	qPCR-Bio-Rad CFX Maestro 1.0, BD FACSCanto™ II (BD Biosciences), Bio-Rad Gel Doc XR+
Data analysis	Microsoft Office Excel 2019 ProPlus, GraphPad Prism 9.0, BD CellQuest™ software version 5.1, Quantity One v4.6, ImageJ

For manuscripts utilizing custom algorithms or software that are central to the research but not yet described in published literature, software must be made available to editors and reviewers. We strongly encourage code deposition in a community repository (e.g. GitHub). See the Nature Portfolio guidelines for submitting code & software for further information.

Data

Policy information about availability of data

All manuscripts must include a data availability statement. This statement should provide the following information, where applicable:

- Accession codes, unique identifiers, or web links for publicly available datasets
- A description of any restrictions on data availability
- For clinical datasets or third party data, please ensure that the statement adheres to our policy

The datasets generated and analyzed during the current study are available from the corresponding author, Dr. Xiaolei Zhou (email address: foxlei@live.cn), upon reasonable request, as described in the following figshare metadata record: https://10.6084/m9.figshare.17088764. The full sequences of the newly constructed plasmids for this study are deposited in Addgene under the ID numbers listed in Supplementary table 1.

Field-specific reporting

Please select the one below that is the best fit for your research. If you are not sure, read the appropriate sections before making your selection.

☒ Life sciences ☐ Behavioural & social sciences ☐ Ecological, evolutionary & environmental sciences

For a reference copy of the document with all sections, see nature.com/documents/nr-reporting-summary-flat.pdf

Life sciences study design

All studies must disclose on these points even when the disclosure is negative.

Sample size	For in vitro study, all the experiments were repeated at least three independent times with technical triplicates. For in vivo study, the sample size were estimated based on preliminary data or for statistical power.
Data exclusions	No data were excluded.
Replication	Replicate experiments were successful.
Randomization	For in vitro study, cells for different groups were treated randomized. For in vivo study, animal injected with cells were chosen randomly.
Blinding	Blinding was not relevant to this study because no bias could be made by the subject or the tester in the experiments performed.

Reporting for specific materials, systems and methods

We require information from authors about some types of materials, experimental systems and methods used in many studies. Here, indicate whether each material, system or method listed is relevant to your study. If you are not sure if a list item applies to your research, read the appropriate section before selecting a response.

Materials & experimental systems

n/a	Involved in the study	
☐	☒	Antibodies
☐	☒	Eukaryotic cell lines
☒	☐	Palaeontology and archaeology
☐	☒	Animals and other organisms
☒	☐	Human research participants
☒	☐	Clinical data
☒	☐	Dual use research of concern

Methods

n/a	Involved in the study	
☒	☐	ChIP-seq
☐	☒	Flow cytometry
☒	☐	MRI-based neuroimaging

Antibodies

Antibodies used	NTI-β-actin (clone AC-74) Mouse mAb (Sigma-Aldrich (St. Louis, MO, USA) Cat# A2228, RRID: AB_476697) ANTI-FLAG® M2 Mouse mAb (Sigma-Aldrich (St. Louis, MO, USA) Cat# F1804, RRID: AB_262044) Anti-HBXIP (H-5) Mouse mAb (Santa Cruz Biotechnology Cat# sc-373980, RRID: AB_10918789) Anti-NRF2 (D1Z9C) XP® Rabbit mAb Cell (Signaling Technology （Danvers, MA, USA） Cat# 12721, RRID: AB_2715528) Rabbit (DA1E) mAb IgG XP® Isotype Control (Cell Signaling Technology （Danvers, MA, USA） Cat# 3900, RRID: AB_1550038) Anti-Caspase-3 (3G2) Mouse mAb (Cell Signaling Technology （Danvers, MA, USA） Cat# 9668, RRID: AB_2069870) Anti-PARP (46D11) Rabbit mAb (Cell Signaling Technology （Danvers, MA, USA） Cat# 9532, RRID: AB_659884) Anti-Lamin B1 (D9V6H) Rabbit mAb (Cell Signaling Technology （Danvers, MA, USA） Cat# 15068, RRID: AB_2798695) Anti-GST3/GSTpi Goat pAb (Abcam (Cambridge, MA) Cat# ab53943, RRID: AB_873819) Goat IgG Isotype Control (Invitrogen, Thermo Fisher Scientific, USA Cat# 02-6202, RRID: AB_2532946) Anti-JNK1 Rabbit mAb (Abcam (Cambridge, MA) Cat# ab110724, RRID: AB_10866293) Anti-phospho-JNK1 Rabbit mAb (Abcam (Cambridge, MA) Cat# ab46821, RRID: AB_881487) Anti-Peroxiredoxin 1 Mouse mAb (R&D Systems （Minneapolis, MN, USA） Cat# MAB3488, RRID: AB_2170319) Anti-E6AP Rabbit pAb (Abcam (Cambridge, MA) Cat# ab3519, RRID: AB_303868) Anti-Human Ubiquitin Rabbit pAb (R&D Systems （Minneapolis, MN, USA） Cat# A-100, RRID: AB_10971295)
Validation	Validations are based on the datasheets from the manufacturers.

Eukaryotic cell lines

Policy information about cell lines

Cell line source(s)	MCF-7 (Cat# HTB-22TM, Lot# 60012672, RRID: CVCL_0031), MDA-MB-436 (Cat# HTB-130, Lot# 61331460， RRID: CVCL_0623), HEK293 (Cat# CRL-1573™, Lot# 7681666， RRID: CVCL_0045) and HEK293T (Cat# CRL-3216, RRID: CVCL_0063) were obtained from ATCC; Nrf2-/- MEF lines were obtained from Tohoku University (Japan); Other stable cell lines were

	constructed in laboratory of Xiaolei Zhou.
Authentication	None of the cell lines used were authenticated.
Mycoplasma contamination	All cell lines tested negative for mycoplasma contamination.
Commonly misidentified lines (See ICLAC register)	No commonly misidentified cell lines were used.

Animals and other organisms

Policy information about studies involving animals; ARRIVE guidelines recommended for reporting animal research

Laboratory animals	female Balb/c mice, 6 weeks old.
Wild animals	The study did not involve wild animals.
Field-collected samples	The study did not involve samples collected from the field.
Ethics oversight	All the experiments involving animals complied with protocols approved by the Institutional Animal Care and Use Committee of the Hebei University of Science and Technology.

Note that full information on the approval of the study protocol must also be provided in the manuscript.

Flow Cytometry

Plots

Confirm that:

☒ The axis labels state the marker and fluorochrome used (e.g. CD4-FITC).

☒ The axis scales are clearly visible. Include numbers along axes only for bottom left plot of group (a 'group' is an analysis of identical markers).

☒ All plots are contour plots with outliers or pseudocolor plots.

☒ A numerical value for number of cells or percentage (with statistics) is provided.

Methodology

Sample preparation	Data provided in the manuscript.
Instrument	BD FACSCanto™ II (BD Biosciences)
Software	BD CellQuest™ software (version 5.1; BD Biosciences)
Cell population abundance	All the samples analyzed by flow cytometry were cell lines with different treatments. For each run, 5000 cells were analyzed based on previous experience.
Gating strategy	FSC/SSC gates were done to exclude cell debris. The boundary between positive and negative staining cells was PE>10

☒ Tick this box to confirm that a figure exemplifying the gating strategy is provided in the Supplementary Information.
